# Taxonomic revision of *Rochefortia* Sw. (Ehretiaceae, Boraginales)

**DOI:** 10.3897/BDJ.4.e7720

**Published:** 2016-06-08

**Authors:** Ramona-Elena Irimia, Marc Gottschling

**Affiliations:** ‡ NIRDBS/Stejarul Research Centre for Biological Sciences, Piatra Neamţ, Romania; § Faculty of Biology, Alexandru Ioan Cuza University of Iaşi, Iaşi, Romania; | Department Biologie, Systematische Botanik und Mykologie, GeoBio-Center, Ludwig-Maximilians-Universität München, München, Germany

**Keywords:** asterids, Caribbean, herbarium specimens, morphology, taxonomy

## Abstract

**Background:**

*Rochefortia* is a small taxon of woody plants in the Ehretiaceae (Boraginales) exhibiting coriaceous leaves with cystoliths, small whitish flowers and drupaceous fruits containing four pyrenes. It shares the dioecious sex distribution with its sister group *Lepidocordia* and can be delimited from the latter (and all other Ehretiaceae) by the presence of thorns. Neotropical *Rochefortia* is distributed over most Caribbean islands, Central America and northern South America. Twenty-eight validly published names (corresponding to twenty-one typified taxa at the species level and below) are available in *Rochefortia*, but the precise number of species to be accepted has been elusive before this revision.

**New information:**

In the course of the present revision, 353 herbarium collections, comprising approximately 540 *Rochefortia* specimens, were entried into a BRAHMS data base providing information about protologues and types and retrospective georeferences if possible. Based on the combination of molecular and morphological data we propose to recognise nine species of *Rochefortia*, namely *R. acanthophora*, *R. bahamensis*, *R. barloventensis*, *R. cubensis*, *R. cuneata*, *R. lundellii*, *R. oblongata*, *R. spinosa* and *R. stellata* (the remaining nineteen validly published names are synonymised under such names). Morphological description of each species and an identification key are provided.

## Introduction

### Taxonomic history

*Rochefortia* Sw. was described by the Swede Olof P. Swartz (1760–1818) in 1788 based on plants he collected in Jamaica. The taxon received its name in commemoration of a French author and priest, namely Charles de Rochefort (1605–1683), who lived in the Caribbean for a period of time and wrote the disputed book “Histoire naturelle et morale des Îles Antilles de l'Amérique ... avec un vocabulaire Caraïbe” (firstly published in 1658). Swartz (1788) included two species, namely *R. cuneata* Sw. and *R. ovata* Sw., the first of which is designated as the type of *Rochefortia* (Britton and Millspaugh 1920). Later, Don (1838) introduced the name *Lutrostylis* G.Don [referring to *Ehretia fasciculata* Kunth, = *R. spinosa* (Jacq.) Urb.] and Triana (1854) the (not validly published: ICN Art. 53.1.) name *Diplostylis* H.Karst. & Triana (based on material he collected together with Gustav K.W.H. Karsten). Both names refer to floral traits that the authors considered unusual for other taxa today assigned to Ehretiaceae. In their descriptions, the authors highlighted the formation of two stylodia, which in fact is characteristic for species of *Rochefortia* such as *R. spinosa*. Subsequently, both names *Diplostylis* (though not validly published) and *Lutrostylis* are sunk in synonymy under *Rochefortia* since Bentham and Hooker (1876).

Twenty-eight validly published names (corresponding to twenty-one typified taxa at the species level and below) are available in *Rochefortia*, but it has been unclear before this revision how many species are to be accepted. The only morphological revision considering the entirety of *Rochefortia* is provided by a Master thesis of Lefor (1968), who recognised five species based on morphology, namely *R. acanthophora* (DC.) Griseb. (considered as widely distributed in the Caribbean), *R. lundellii* Camp (as Mexican endemic), *R. oblongata* Urb. & Ekman (as eastern Cuban endemic), *R. stellata* Britton & P.Wilson (as eastern Cuban endemic) and *R. spinosa* (as widely distributed in the Caribbean and South America). He included the type species *R. cuneata* (Jamaica) in the latter because of lacking diagnostic characters. Another attempt to shed light on the taxonomy of *Rochefortia* is the conspectus of Klotz (1982), who accepted eleven species. However, he failed to provide a key or descriptions and therefore, delimitation of species remains rather obscure. Klotz (1982) subdivided *Rochefortia* into two sections, *Rochefortia* and *Stellatae* G.Klotz (featuring the species with multi-branched trichomes: Klotz 1980), as well as Rochefortia sect. Rochefortia into two series, *Rochefortiae* and *Acanthophorae* G.Klotz.

During the 20^th^ century, most taxonomic treatment of *Rochefortia* was performed for restricted geographical regions only (Cuba: Sauget y Barbis [Léon] and Liogier de Sereys Allut [Alain] 1957; Jamaica: Adams 1972; Dominican Republic and Haiti: Liogier de Sereys Allut 1994; Puerto Rico: Little Jr et al. 1988, Liogier de Sereys Allut 1995, Liogier de Sereys Allut and Martorell 1999; Belize: Dwyer and Spellman 1981; Guatemala: Standley and Williams 1970). A notable contribution was the careful revision of Cuban taxa assigned to *Rochefortia*: Klotz (1979) payed much attention to apparently different inflorescence types, but later also to leaf size, shape and indument in order to distinguish species (Klotz 1980). His work is summarised with the description of seven new taxa (four at the species level and three below) although in specific cases, he was unsure himself whether such variation is in fact established in reproductively isolated units, or is rather expression of ecological modification (Klotz 1980). Overall, few illustrations of *Rochefortia* (e.g., *Rochefortia*: Swartz 1797; *R. acanthophora*: Gürke 1893, Liogier de Sereys Allut 1995;*Gürke 1893R. barloventensis* Irimia & Gottschling: Irimia and Gottschling 2015; *R. lundellii*: Standley and Williams 1970; *R. spinosa*: Jacquin 1781) or field images of living plants (e.g., *R. acanthophora*: Moser et al. 2010) were published to date.

### Morphology

**HABIT**. *Rochefortia* comprises evergreen shrubs or trees 1.5 m up to 10.0 m tall (*R. spinosa*) or even lianas (*R. lundellii*). In *R. lundellii*, *R. spinosa* and to some extent also in *R. acanthophora*, twigs are arching, and the stem is divided right from the base like in a shrub (herbarium specimen notes). The bark is light grey through brown in colour and its surface smooth or slightly fissured, sometimes peeling in flakes (*R. acanthophora*). The young twigs and leaves are variously tomentose (*R. stellata*) through scabrous (*R. acanthophora*, *R. cubensis* Britton & P.Wilson, *R. oblongata*) and sometimes displaying only a few scattered trichomes congregating on both the adaxial and abaxial leaf midrib (*R. bahamensis* Britton, *R. lundellii*, *R. spinosa*).

All species of *Rochefortia* develop thorns being a unique and therefore diagnostic character in Ehretiaceae (Gottschling et al. 2014b, Gottschling et al. 2016). Thorns in *Rochefortia* derivate either from bracts (when fasciculate leaves are developed in their axils) or from brachyblasts (i.e., short shoots; Urban 1908, Lefor 1968, Klotz 1979). They are predominantly simple in *Rochefortia*, but dichotomously branched thorns are present in Cuban and Jamaican species (i.e., *R. cubensis*, *R. oblongata*, *R. stellata*). Thorns occur particularly in those parts of the plants, where reproductive organs are produced (Klotz 1979, Klotz 1980). In *R. lundellii*, the fully developed stems also bear thorns (as inferred from herbarium specimen notes).

**Figure 1.**
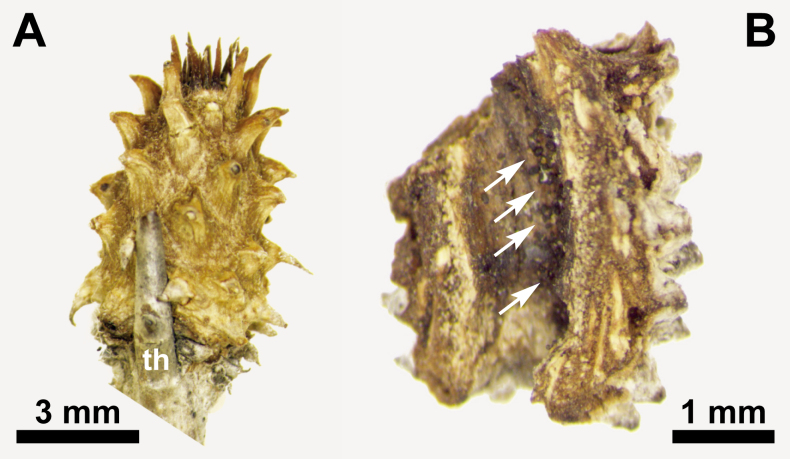
**Galls**. Cuban species occasionally exhibit internally hollow short shoots composed of rudimentary leaves in axils of (modified thorny) bracts or brachyblasts. A. general gall morphology of *R. oblongata*; B. longisection of gall, *R. cubensis*, arrow indicate reproductive structures putatively of mites.

In Cuban species such as *R. cubensis* and *R. oblongata*, short shoot structures are developed (Fig. 1) that are internally hollow. They originate in the axil of a thorn or occasionally of a leaf (i.e., where tissue is soft and injectable) and are composed of multiple reduced leaves. No intermediate structures between the strobili and mature plant parts such as leaf clusters or inflorescences have been observed in the course of this revision. To the best of our knowledge, they have not been noted before in *Rochefortia*, and we interpret such structures as galls developed after induction of insects such as mites.

**LEAVES (Fig. 2)**. Phyllotaxis in mature parts of the plants is predominantly fasciculate, but is sometimes alternate or rarely subopposite in the big-leaved mainland species (i.e., *R. lundellii*, *R. spinosa*) and to some extent also in *R. cuneata* from Jamaica. The length of the petiole is variable ranging from 0.05–0.2 cm in *R. acanthophora* and *R. cubensis* to 0.3–0.5 cm in *R. stellata* and considerably longer (1.5–2.0 cm) in the species with intermediate sized and large leaves (i.e., *R. bahamensis*, *R. barloventensis*, *R. cuneata*, *R. lundellii*, *R. spinosa*).

Leaf texture is usually coriaceous and occasionally membranaceous (individuals of *R. cuneata*). The most common leaf shapes in (woody Boraginales such as) *Rochefortia* are elliptic and obovate, some individuals in *R. bahamensis* and *R. barloventensis* additionally have nearly spherical leaves. In general, plants with larger leaves (i.e., >5.0 cm) assigned to *R. cuneata*, *R. lundellii* and *R. spinosa* are rather easily distinguishable from plants with smaller leaves (i.e., <5.0 cm) present in, for example, *R. acanthophora* and *R. bahamensis*. *Rochefortia cubensis* is the species with the smallest leaves (1.0 cm maximal length), while *R. lundellii* and *R. spinosa* are at opposite extremes with leaf lengths up to 12.0 cm. The lamina is always simple, with the margin entire or sometimes revolute (e.g., *R. cuneata*) and with the tips ciliate. The leaf base is cuneate or rounded, while the apex is rounded through retuse, acute or sometimes cuspidate (*R. lundellii* and also a few individuals of *R. spinosa*).

Leaf indument usually comprises at least few scattered trichomes at midribs, but leaves and individuals being completely glabrous are occasionally found in *R. bahamensis*, *R. barloventensis*, *R. cuneata*, *R. lundellii* and *R. spinosa*. The presence of a more extensive indument leading to a, for example, hirsute surface of leaves is likewise rare (e.g., *R. cubensis*, *R. stellata*). Trichomes are predominantly unicellular and simple, with the exception of *R. stellata* having characteristic multi-branched and star-shaped trichomes (Fig. 2). Usually, the trichomes on the abaxial leave surface (sometimes also on the adaxial surface) causing the typical roughness of the leaf surface contain cystoliths (that are present also in adjacent subsidiary cells). Function of such cystoliths remains rather elusive at present and may include either excretion or repellant or defence against herbivores. On a single herbarium specimen (Lasser 758: US!) determined as *R. spinosa*, two deciduous stipules are noted that are generally rare in Ehretiaceae (Gottschling et al. 2016).

**FLOWERS**. Generative organs are rarely documented in herbarium specimens, whereas knowledge about flowers and fruits are particularly scarce for Cuban species of *Rochefortia*. The basic monotelic architecture being thyrsoid shows little variation in *Rochefortia*, and inflorescences are positioned both axillarily and terminally. The number of flowers in each inflorescence usually varies between 3–10, but can be increased up to 15 (*R. lundellii*, *R. spinosa*) or can be reduced to a cluster of 2 and sometimes to a single flower (*R. acanthophora*, *R. cubensis*). In inflorescences, bracts are absent in *Rochefortia*, and flowers are pedicellate through subsessile. The latter trait has taxonomic importance to delimit, for example, *R. acanthophora* (with sessile flowers) from morphologically similar species (such as *R. cubensis* with shortly pedicellate flowers).

In *Rochefortia*, flowers are actinomorphic, tetracyclic, pentamerous and unisexual. Most species have small flowers with corolla diameter of about 0.40–0.50 cm, while *R. bahamensis* has significantly larger flowers up to 0.70 cm in corolla diameter (♂ individuals). The synsepalous calyx is coriaceous and its aestivation imbricate (plesiomorphic condition in Ehretiaceae: Gottschling 2004). The calyx tube is short, and the 5 (or rarely 4 or 6) lobes are obovate or triangular (*R. stellata*), with an acute apex. The outer surface is hirsute and ciliate at tips, whereas the inner surface is glabrous and sometimes, a few scattered trichomes are present towards the distal part. The sympetalous corolla is membranaceous and its tube funnel-shaped, occasionally 4 or 6 instead of usually 5 lobes are present. Both corolla surfaces are glabrous, somewhat rugose, and slightly ciliate at tips. Corolla colour is usually white or yellow, occasionally whitish green.

Female and male flowers differ only with respect to formation of gynoecium and androecium, but not to perianth. Male flowers have very well developed stamens with (in outline) reniform functional anthers as demonstrated by the presence of pollen (visible with the stereo microscope at 60x magnification). Filaments are flattened, which is another unusual character within Ehretiaceae (Lefor 1968). The ovary in male flowers is conical, choricarpous to symplicate at the base and proceeds apically into two rudimentary stylodia (Irimia and Gottschling 2015). The adaxial surface of the stylodia is strigose. Overall, morphological variation in male flowers is low between species of *Rochefortia*.

Female flowers have smaller, shrivelled anthers lacking pollen (verified in SEM in the course of the present study) on short filaments. The ovary is globose and exhibits the coenocarpous-syncarpous architecture usually developed in Ehretiaceae (Gottschling 2004, Gottschling et al. 2014a). The style is bifid, whereas the branches divide in the proximate half (*R. acanthophora*, *R. barloventensis*, *R. cuneata*, *R. lundellii*, *R. stellata*), or two stylodia are developed (*R. cubensis*, *R. spinosa*). Style length varies from shorter (*R. stellata*) through longer than the diameter of the prospective fruit (*R. acanthophora*, *R. barloventensis*, *R. cubensis*, *R. lundellii*, *R. spinosa*). The entire gynoeceal structure occasionally is spangled with few scattered trichomes (e.g., *R. stellata*). The two stigmas are capitate through cotyliform and usually are very extensive.

Sex distribution is dioecious, which is a very unusual trait within Ehretiaceae (and argues as synapomorphy for the close relationship between *Lepidocordia* and *Rochefortia*: Miller and Nowicke 1990, Gottschling et al. 2014b). However, data basis for flowering times is poor for most species of *Rochefortia*. The more specimens are available, the greater is the number of months, in which both sexes occur at the same time (e.g., 4 months in *R. acanthophora*, 3 months in *R. spinosa*). The impression is even stronger when data of all species are pooled over the year (Table 1) and thus, there is no such thing as a temporal separation of the sexes in *Rochefortia* as indicated by Miller and Nowicke (1990) for *Lepidocordia*.

Flowers are sometimes fragrant (as noted on herbarium specimens) indicating zoophily like in many other Ehretiaceae. There is a single report about pollinators available from herbarium collections (Zanoni et al. 39552: MO!) stating that flowers are visited by numerous bees (species not indicated).

**FRUITS**. Fruit is indehiscent and drupaceous, with the shape (sub-)spherical and a diameter of 0.60–0.70 cm. Individuals of *R. lundellii* from Costa Rica exhibt the largest fruits at all found in *Rochefortia* with a diameter of 1.00–1.20 cm. The inner architecture of the fruit, and particularly of the endocarp, corresponds (almost indistinguishable) to the four-parted type known from the *Ehretia* P.Browne I clade (Ehretiaceae; Pitot 1939, Verdcourt 1991, Gottschling and Hilger 2001). The abaxial surface of the pyrenes varies from smooth and plain (*R. oblongata*) through reticulately ornamented with ridges in most other species such as *R. spinosa* (Miller 1989). The seed is bent and surrounds extensive placental tissue that is not enclosed in an individual sterile chamber of the pyrene (as it is the case in *Bourreria*: Gottschling 2004, likewise from Ehretiaceae).

### Distribution and habitat

Most *Rochefortia* species grow in dry, only seasonally wet climate, relatively close to the sea and at low altitudes. Preferred substrates include rocky and alkaline soils with a calcareous layer, and most of the West Indian species are reported to occur on both limestone and serpentine. Species of *Rochefortia* are important elements of xeromorphic plant communities that are notedly characteristic in Cuba and include large portions of endemics (Borhidi 1991). Somewhat deviating from the other species of *Rochefortia*, Central American *R. lundellii* and South American *R. spinosa* are abundant in wet forests at higher altitudes.

**Figure 3.**
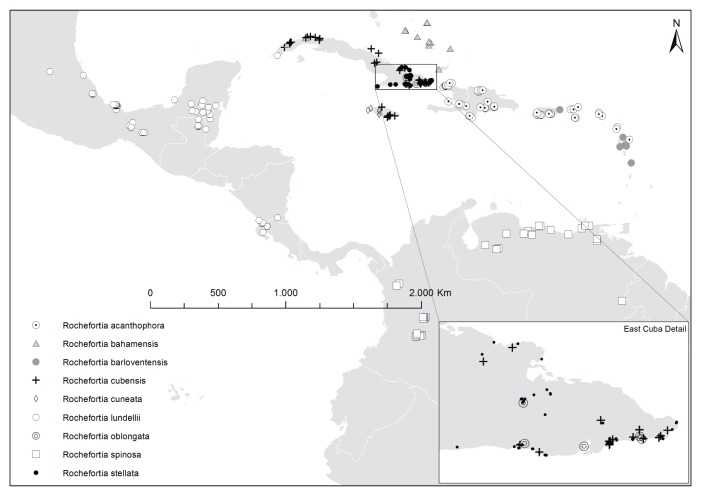
**Circumcaribbean distribution of *Rochefortia***. Note that many species of *Rochefortia* are delimited geographically and that *R. acanthophora*, for example, has a more restricted range on the Greater Antilles (i.e., is absent from Cuba and Jamaica) than previously considered.

*Rochefortia* is restricted to Caribbean islands and the adjacent American mainland (Fig. 3), with a putative distribution gap in Panama, from where no collection has been reported to date. Cuba is the centre of diversity with four native species (i.e., *R. bahamensis* in western Cuba, widely distributed *R. cubensis* and two endemics, *R. oblongata* and *R. stellata*, from eastern Cuba) as well as a putative neophyte (i.e., *R. lundellii*). The other Greater Antilles harbour maximally two species each (Jamaica: *R. cubensis*, *R. cuneata*; Hispaniola: abundant *R. acanthophora*, rare *R. cuneata* as tentative determination; Puerto Rico: abundant *R. acanthophora*, rare *R. barloventensis*). Everywhere else, *Rochefortia* occurs with a single species (Central America: *R. lundellii*; northern South America: *R. spinosa*; Lesser Antilles: *R. barloventensis* [except Guadeloupe, where *R. acanthophora* has been also documented: Howard 1989], The Bahamas: *R. bahamensis*). Overall, a strong biogeographic signal can thus be inferred from the distribution of *Rochefortia* species, and geographic occurrence rather than morphology is indicative for species determination (Irimia et al. 2015).

## Materials and methods

Delimitation of *Rochefortia* species was inconsistent among previous authors (Lefor 1968, Klotz 1980, Klotz 1982) because of a high degree of variability and plasticity of morphological traits. Since then, there are significantly more collections available in the herbaria of the world than were studied before, and this allowed for a comprehensive review of the morphological variability over the entire geographic range of *Rochefortia*. Our species circumscriptions are based on two major sources of information, namely 1) molecular phylogenetics comprising an exhaustive taxon sample over the entire morphological variation and geographical range of *Rochefortia* populations (Irimia et al. 2015) and 2) morphological investigations of 353 herbarium collections comprising approximately 540 *Rochefortia* specimens.

Species of *Rochefortia* exhibit a clear biogeographic correlation (Fig. 3) as inferred from molecular sequence data (Irimia et al. 2015). The Caribbean group comprising 7 species constitutes the sister group of the two joint mainland species. Within the Caribbean species group, *R. barloventensis* is largely restricted to the Lesser Antilles (Irimia and Gottschling 2015), whereas *R. acanthophora* mainly occurs on the eastern Greater Antilles. The remaining 5 species are distributed over The Bahamas, Cuba and Jamaica (but phylogenetic resolution within that clade is poor). This basic scaffold provided by molecular sequence data was used to re-evaluate morphological variation documented in numerous specimens of *Rochefortia*.

We adopted a rather broad morphological species concept for *Rochefortia* and concluded that sympatric populations of different species should not be difficult to distinguish and should not include morphological intermediates. Basically, we started our revision by addressing the question how many species can be distinguished on every separate island in the Caribbean as center of diversity. As a result, we now recognise 9 species in *Rochefortia* having distributions that are more or less contiguous. However, the island species (e.g., *R. bahamensis*, *R. barloventensis*, *R. cubensis*), but also Central American *R. lundellii*, exhibit some degree of disjunction in their distributions.

Different from many other taxa of woody borages, species of *Rochefortia* lack exclusive characters with respect to generative organs. Using such traits for diagnostic purposes is further impeded since they are rarely documented on herbarium specimens: Already Lefor (1968) and Klotz (1979) complained about the large amount of sterile among *Rochefortia* specimens. As a result, intraspecific variability of generative organs is continuously not well worked out for species such as *R. cubensis, R*. *oblongata* and *R. stellata*, which remains a challenging future task. Anyhow, length of pedicles, number of flowers per inflorescence and division and total length of styles show considerable variation and appear partly useful to recognise and delimit species as inferred from generative organs.

Vegetative traits help to distinguish between species that occur sympatrically, but the degree of homoplasy is high when considered over the entire geographic range. Individual specimens of, for example, *R. bahamensis* and *R. barloventensis* can be remarkably similar in their morphology, although no similar morphologies are found within the geographic distance of more than 500 km. The only highly distinctive trait among species of *Rochefortia* is the presence of stellate trichomes on leaf surface and other plant parts, which is diagnostic for the Cuban endemic *R. stellata* (Lefor 1968, Klotz 1982). Moreover, plants with larger leaves (i.e., >5.0 cm) assigned to *R. cuneata*, *R. lundellii* and *R. spinosa* may be easily distinguishable from plants with smaller leaves (i.e., <5.0 cm) determined as, for example, *R. acanthophora*, *R. bahamensis* and *R. cubensis*.

In this revision, we provide comprehensive nomenclatural data about all names published for species of *Rochefortia*. They are linked to online resources such as Biodiversity Heritage Library (BHL) and Biodiversity Literature Repository (BLR) for bibliography (i.e., protologues) and JSTOR for high quality images of type specimens (although free access is limited). The present printed revision of *Rochefortia* is also part of an ongoing online project on the dedicated platform for cyber-taxonomy, namely Botanical Research and Herbarium Management System (BRAHMS). We thus made our BRAHMS data base publicly available, which provides access to the information implemented in hundreds of *Rochefortia* specimens. Searches by taxon, collector, locality name and map area (Fig. 3) are possible and generate tables that can also be shown in text or excel format. The flexibility of the BRAHMS platform will enable us also to upload more images, individual observations and future studies of *Rochefortia* in a comprehensible way. We thus aim at an improved biodiversity knowledge about a largely neglected branch of Ehretiaceae.

## Taxon treatments

### Rochefortia

Sw.

Rochefortia ***Rochefortia*** Sw., Prod.: 53. 1788, Fl. Ind. Occid. 551, pl. XI. 1797. Rochefortia sect. Stellatae G.Klotz, Wiss. Z. Friedrich-Schiller-Univ. Jena, Math.-Naturwiss. Reihe 29: 470. 1980. Rochefortia ser. Acanthophorae G.Klotz, Revista Jard. Bot. Nac. Univ. Habana 3: 105. 1982.
Rochefortia = *Lutrostylis* G.Don, Gen. hist. 4: 391. 1838.—TYPE: *Lutrostylis spinosa* (Jacq.) G.Don, designated here. Don (1838) included three species in his *Lutrostylis*, of which *L. inermis* G.Don (though not validly published: ICN Art. 52.1., ≡ *Ehretia fasciculata* Kunth) and *L. spinosa* are heterotypic synonyms of *R. spinosa*, while *L. montevidensis* (Spreng.) G.Don belongs to Verbenaceae.
Rochefortia Rochefortia cuneata Sw.Britton and Millspaugh (1920) Reason for typification: Indication or designation of a type in the protologue, names of genera or subdivisions of genera (Art. 10, 40).

#### Description

Shrubs or trees, rarely lianas, thorny, short shoot galls present or absent; bark striate, with superficial grooves, longitudinally fissured, grey light to dark brown, with lenticels; indument absent at maturity or puberulent, trichomes simple, sometimes glandular, rarely branched (*R. stellata*). Leaves fasciculate, rarely alternate or subopposite (*R. lundellii*, *R. spinosa)*, simple; petiole hirsute through glabrous; blade elliptic through obovate, sometimes orbicular (*R. bahamensis*), margin entire, texture coriaceous, occasionally membranaceous (*R. cuneata*), venation brochidodromous, reticulate; indument variously pubescent through hispid, rarely absent, trichomes unicellular, frequently containing cystoliths (also in adjacent subsidiary cells) and causing the typical roughness of leaf surface. Inflorescence thyrsoid, terminal or axillary, branching sympodial, sometimes heavily condensed or flowers solitary, ebracteolate. Buds obovoid, whitish green; flower actinomorphic, unisexual, sometimes fragrant, distinctly pedicellate (*R. bahamensis*, *R. barloventensis*, *R. lundellii*, *R. spinosa*) to sub-sessile (*R. stellata*) or almost sessile (*R. acanthophora, R. cubensis*); calyx persistent, coriaceous, campanulate, aestivation imbricate; corolla sympetalous, predominantly white or rarely yellow orange or greenish, turning brown at later ontogenetic stage, membranaceous, funnel-shaped, aestivation imbricate; stamens 5, rarely 4 or 6, exserted, filaments adnate to corolla tube for 0.10–0.15 cm, flattened, anthers of male flowers with pollen, anthers of female flowers empty and diminutive; ovary of male flowers choricarpous, carpels fused only at the gynobase, stylodia 2, distally with a tuft of dense, grey trichomes, ovary of female flowers coencarpous-syncarpous, with a well developed style consisting of 2 distinct branches or with 2 stylodia, style or stylodia 2–2.5 times longer than ovary and fruit, stigmas capitate, usually well developed. Fruit indehiscent, drupaceous, exocarp brightly red at maturity, later turning brown, exocarp membranaceous, mesocarp fleshy, endocarp ligneous and 4-parted; pyrene smooth through variously ornamented on abaxial surface, enclosing 1 seed, placenta extensive, but not enclosed in sterile chamber of pyrene, embryo curved.

### Rochefortia acanthophora

(DC.) Griseb.

Rochefortia acanthophora ***Rochefortia acanthophora*** (DC.) Griseb., Fl. Brit. W. I.: 482. 1862. *Ehretia acanthophora* DC. in A.DC., Prodr. 9: 510. 1845.—TYPE: Caribbean, Dominican Republic. Without precise locality (1821): C.G.L. Bertero s.n. 1821 (sterile) (lectotype, designated here: G-DC18088! isolectotypes: MO-1886637! P *fide* Lefor 1968); without precise locality at Biajama river (1819): C.G.L. Bertero s.n. 1819 (sterile) (syntype: TO-4998!).

#### Description

Shrubs up to 4.0 m tall, rarely trees 4.0–7.0 m tall, galls absent; twig indument absent or sericeous, glabrescent at maturity, trichomes simple; bark grey whitish to grey dark or brown, sometimes peeling; thorns 0.5–0.8 cm long, slender, acute, simple, numerous, axillary, alternate, glabrous through variously sericeous. Leaves fasciculate; petiole 0.05–0.2 cm long, slender, hirsute; blade 0.5–1.5 cm long, 0.2–0.3 cm wide, elliptic to obovate, widely obovate or circular (particularly distal immature leaves), coriaceous, primary vein prominent, with scattered trichomes, secondary veins 3–6, tertiary veins arcuate or reticulate; base cuneate, rarely rounded; apex rounded or obcordate, occasionally retuse, emarginated or cleft; adaxial surface hirsute to pubescent, with simple trichomes and cystolith-like structures in subsidiary cells, abaxial surface bright, variously hirsute to glabrous. Inflorescence axillary, flowers in clusters of 2 or rarely 3 or solitary, pedicels up to 0.02-0.03 cm long. Calyx 0.40–0.50 cm long, 0.45–0.50 cm wide, coriaceous, glabrous or with few scattered trichomes outside, sometimes hirsute, glabrous inside with a tuft of trichomes at the distal part of lobes, lobes 0.35–0.40 cm long, 0.40–0.43 cm wide, ovate to broadly ovate, apex acute, margin strigose. Corolla 0.40–0.50 cm long, white, yellowish or pale yellow, rarely green (as in Leonard & Leonard 1579: GH!, Leonard & Leonard 2579: US!, Leonard & Leonard 12579: GH! US!), brownish when dry, membranaceous, glabrous on both surfaces, tube 0.20–0.25 cm long, funnel-shaped, lobes 0.35–0.40 cm long, ovate, rounded, margin slightly ciliate. Anthers of male flower 0.50–0.55 cm long, oblong, filaments 0.45–0.50 cm long, adnate to the corolla tube for further 0.10–0.15 cm, pollen present (SEM), tricolporate; anthers of female flower 0.25–0.30 cm long, oblong, pollen absent, filaments very short, 0.05–0.07 cm long, adnate to the corolla tube for further 0.10–0.12 cm. Ovary of male flower subglobose, 0.10–0.20 cm long, stylodia 2, 0.10–0.15 cm long, distally setose, with grey trichomes, ovules absent; ovary of female flower globose, style 0.35–0.40 cm long, divided, branch 0.30–0.35 cm long, stigmas very well developed, cotyliform. Fruit 0.30–0.45 cm tall, 0.40–0.50 cm wide, globose, green when immature, later orange brown or red to brightly red at maturity, brown when old, mesocarp fleshy; pyrene 0.30–0.40 cm tall, 0.30–0.35 cm wide, 0.35–0.40 cm deep, abaxial surface with reticulate ridges.

#### Distribution

Coastal habitats and scrublands across Caribbean islands between Haiti in the West and Guadeloupe in the East, with high abundance in the Dominican Republic, Puerto Rico and the Virgin Islands (symbol "⨀" in Fig. 3), at low altitudes (sea level up to 300 m), occasionally at higher altitudes of 1200 m (Jiménez 4192: US!). Plant species with similarly restricted distributions on the eastern Greater Antilles are otherwise rare (4 taxa at the generic level listed in Borhidi 1991).

#### Ecology

Flowering throughout the year (Jan–Dec); fruiting Oct–Mar, May–Aug, but probably contiguously.

#### Taxon discussion

Traditionally, *R. acanthophora* has been considered the most widely distributed species of *Rochefortia* in the Caribbean (Lefor 1968), but the results from a molecular survey rather indicate a restricted geographic range (Irimia et al. 2015). Particularly, *R. acanthophora* has its westernmost distribution in Haiti, but is absent from Cuba (firstly noted by Klotz 1982 because of leaf shape differences) and Jamaica, where it has been believed to occur in the past. Anyhow, the species is well represented on the islands where it occurs, and is the only small-leaved species documented for Haiti and Dominican Republic, Puerto Rico, British as well as US Virgin Islands and Antigua.

*Rochefortia acanthophora* is sympatric with only a single other species, namely *R. barloventensis*, from which it differs in the frequently axillary (*versus* predominantly terminal) inflorescences comprising few (and not more than 5) flowers, a smaller leaf blade size (0.5–1.5 cm long *versus* 1.4–3.4 cm long) and a reduced leaf petiole length (0.05–0.2 cm long *versus* 0.4–1.5 cm long). Anyhow, sympatry between both species is restricted to Guadeloupe (Howard 1989) and Puerto Rico (Irimia et al. 2015).

Morphologically, *R. acanthophora* is a variable species, particularly with respect to leaf size and shape, and exhibits some overlap to *R. cubensis* from Cuba and Jamaica (it was occasionally hard to determine the species reliably if geographic origin was unknown). Among the remaining small-leaved species, it differs from *R. bahamensis* in the more predominantly obovate (*versus* circular) leaf shapes and from *R. stellata* in the absence (*versus* presence) of multi-branched trichomes. Some individuals of *R. acanthophora* (e.g., Liogier 20494: F!, Leonard & Leonard 12760: US!, Ekman 2154: A! F!) appear as substrate for plants assigned to *Tillandsia* sp. (Bromeliaceae).

#### Notes

Representative specimens examined. — HAITI. **Port-de-Paix**: Baie des Moustiques: trail W to main road, vicinity of Cabaret, 19°52’N, 73°03’W [retroactively inferred], 12 Jan 1929 (♀ fl, fr), Leonard & Leonard 11790 (GH! US!); Jean-Rabel: along Mole Road, 19°51’N, 73°11’W [retroactively inferred], 29 Jan 1929 (♀ fl, fr), Leonard & Leonard 12619 (US!); E of Bord-de-Mer, 19°53’N, 73°12’W [retroactively inferred], 1 Mar 1929 (♀ fl, fr), Leonard & Leonard 13619 (US!); **Île de la Gonâve**: Mahautiere, 18°50’N, 73°14’W [retroactively inferred], 11 Aug 1927 (fr), Ekman 8883 (G! GH!); **Port-au-Prince**: Plaine du Cul de Sac: bw Sources Matelas and Source Puantes, 18°41’N, 72°17’W (retroactively inferred), 15 Oct 1924 (♀ fl, fr), Ekman 2154 (A! F!). — DOMINICAN REPUBLIC. **Azua**: km 82 on road bw Baní and Azua, 18°24’N, 70°33’W [retroactively inferred], elev 50 m, 27 Oct 1973 (♀ fl, fr), Liogier 20494 (F!); **Independencia**: Hoya de Enriquillo: 4 km S of Boca de Cachón on road to Jimaní, 18°31’N, 71°45’W [retroactively inferred], elev at least 30 m, 28 May 1987 (♂ fl), Zanoni et al. 39522 (MO! US!); **Pedernales**: Cerca de Cabo Rojo, 17°54’N, 71°36’W [retroactively inferred], elev 100 m, Jun 1975 (fr), Liogier & Liogier 23240 (GH! NY!); **Santo Domingo**: Trejo, 18°27’N, 69°58’W [retroactively inferred], 1944 (♀ fl, fr), Schiffino 165 (GH!); **Santiago**: Las Lavas, about 25 miles NW of Santiago, 19°28’N, 71°14’W [retroactively inferred], elev 200 m, 6 May 1968 (fl), Liogier 11154 (P! US!). — PUERTO RICO. **Cabo Rojo**: on road bw Cabo Rojo and Pitahaya, 18°63’N, 67°94’W [retroactively inferred], 12 Jul 1963 (♀ fl, fr), Liogier 9912 (F! GH! US!); **Coamo**: vicinity of Coamo Springs, 18°21’N, 66°22’W [retroactively inferred], 10 Feb 1922 (fr), Britton et al. 5987 (F!); bw Serrilos and Salinas, 18°41’N, 66°21’W [retroactively inferred], 9 Dec 1885 (♀ fl, fr), Sintenis 2993 (K! G! GH! M! US!); **Guánica**: Guánica forest, along main road a few meters from entrance to forest, 17°58’N, 66°55’W [retroactively inferred], 7 Oct 1989 (fl), Acevedo-Rodriguez & Chinea 3002 (US!); **Salinas**: rte 712 at rte 706, 18°14’N, 66°13’W [retroactively inferred]), elev ca 200 m, 4 Nov 1989 (fr), Taylor 9592 (NY!); **Lajas**: S of Boquerón 18°25’N, 67°32’W (retroactively inferred), 18 Aug 1950 (♀ fl, fr), Little 13629 (GH!). — BRITISH VIRGIN ISLANDS. **Virgin Gorda**: SE side of peak, 18°29’N, 64°24’W [retroactively inferred], elev ca 150 m, 22 Jun 1969 (♀ fl, fr), Little et al. 23824 (US!). — US VIRGIN ISLANDS. **Buck Island**: Buck Island Reef National Monument, NW end of island, 17°47’N, 64°37’W [retroactively inferred], elev ca 15 m, 8 Apr 1967 (♂ fl), Little 22034 (A! US!); **Saint John**: Reef Bay Quarter; W slope of hill, NE of Fish Bay, 18°19’N, 64°46’W [retroactively inferred], elev 30 m, 10 Jan 1991 (fl), Acevedo-Rodriguez & Siaca 3909 (US!); **Saint Thomas**: Flaghill, 18°19’N, 64°54’W [retroactively inferred], elev ca 180 m, 14 Jun 1876 (♀ fl, fr), Eggers s.n. (GH!). — ANTIGUA: E of Willock Village, 17°95’N, 61°49’W [retroactively inferred], 18 Jun 1937 (fr), Box 858 (GH! GH! US!); Crabhill, 17°15’N, 61°53’W [retroactively inferred], 1849 (fr), Wullschlaegel 384 (W! M!). — SAINT MARTIN. **Dutch St. Martin**, Red Rock, 18°65’N, 63°15’W [retroactively inferred], 9 Apr 2000 (fl), Sastre & Breuil-See 9982 (P!). — FRANCE. **Guadeloupe**: Saint Barthélemy, Gouverneur, 16°19’N, 61°05’W [retroactively inferred], 13 Apr 2000 (fr), Sastre & Breuil-See 10032 (P!).

##### Common names

“bois d’ébène”, “ébénier noir”, “galle-galle”, “gratte-galle” in Haiti, “corazón de paloma” (Span., pigeon heart), “ébano” and “trejo” in Dominican Republic, “juso” in Puerto Rico and “greenheart ebony” in US Virgin Islands (Moser et al. 2010).

##### Economic use

The trunk is employed to make posts for fences (Jiménez 4593: US!), and the wood is reported to be very hard (“bastard lignum vitae”, holly wood: Cook 54: GH!).

### Rochefortia bahamensis

Britton

Rochefortia bahamensis ***Rochefortia bahamensis*** Britton, Bull. New York Bot. Gard. 5: 317. 1907. Rochefortia cuneata subsp. bahamensis (Britton) G.Klotz, Revista Jard. Bot. Nac. Univ. Habana 3: 103. 1982.—TYPE: Caribbean, Commonwealth of The Bahamas. San Salvador [Watling’s] Island, scrub lands near Lighthouse (Mar 13, 1907), N.L. Britton & C.F. Millspaugh 6167 (♀ fl) (holotype: NY: 111152! isotype: F-51163!).

#### Description

Shrubs or small trees up to 1.5–4.5 m tall, galls absent; twig indument puberulent, trichomes simple; bark greyish white to brown, longitudinally fissured, scaly, wood very brittle; thorns 0.3–0.8 cm long, slender, distally somewhat acute, simple, scattered, alternate, axillary or rarely terminal, sericeous. Leaves fasciculate, rarely alternate (Correll 45046: US!, Correll & Wasshausen 46731: US!); petiole 0.5–1.2 cm long, hirsute through pubescent or glabrous; blade 0.7–5.1 cm long, 0.5–2.8 cm wide, obovate, widely obovate or very widely obovate through orbicular (particularly distal immature leaves), coriaceous, primary vein prominent, sometimes with scattered trichomes, secondary veins 3–8, tertiary veins arcuate; base cuneate or rounded; apex rounded, emarginate, obcordate or retuse, rarely cleft; adaxial surface glabrescent, sometimes distally cilliate, bright, with dense cystolith-like structures in epidermal cells, abaxial surface glabrous, occasionally with simple, scattered trichomes, immature leaves sometimes barbellate. Inflorescence axillary, branching sympodial, branches relatively slender, hirsute, pedicels 0.05–0.30 cm long. Calyx 0.35–0.45 cm long, coriaceous, hirsute outside, glabrous inside, lobes 0.35–0.40 cm long, 0.40–0.43 cm wide, divided from the base, ovate to broadly ovate, apex acute, margin strigose, glabrous inside with a tuft of trichomes in upper part of lobes. Corolla 0.50–0.70 cm long, yellow or white-greenish, sometimes fragrant, membranaceous, glabrous on both surfaces, tube 0.20–0.25 cm long, funnel-shaped, lobes 0.50–0.60 cm long, oblong or obtuse, glabrous, rarely with trichomes at margin. Anthers of male flower 0.45–0.50 cm long, oblong, filaments 0.35–0.45 cm long, glabrous, adnate to corolla tube for 0.10–0.12 cm, pollen present, tricolporate; female flower unknown. Ovary of male flower subglobose, 0.15–0.18 cm long, stylodia 2, divided in distal part, branches 0.10–0.12 cm long, hirsute. Fruit 0.40–0.50 cm tall, 0.40–0.60 cm wide, globose, red at maturity; pyrene 0.37–0.40 cm tall, 0.26–0.30 cm wide, 0.12–0.20 cm deep, abaxial surface cutate.

#### Distribution

Across multiple islands of The Bahamas archipelago (Crooked Island, Great Exuma, Inagua, Long Island, Mayaguana, San Salvador) and putatively also in western Cuba (Pinar del Río, Havana) in scrub lands, rocky coppice hills and thicket edges, at sea level or slightly above (symbol "▲" in Fig. 3).

#### Ecology

Flowering Feb–Mar, Oct–Dec; fruiting Dec–Jan, Oct.

#### Taxon discussion

*Rochefortia bahamensis* is an abundant and morphologically very consistent species in The Bahamas archipelago characterised by the predominantly circular leaf shapes. Overall similarity, however, is great with *R. barloventensis* from the Lesser Antilles, but molecular data indicate that the two species are only distantly related (Irimia et al. 2015). The geographical disjunction (> 500 km apart) provides further evidence for the distinctiveness of both species.

The 3 sterile collections from Cuba are tentatively placed under *R. bahamensis* and are morphologically somewhat intermediate between *R. bahamensis* and *R. cubensis*. With *R. bahamensis*, the plants share the more membranaceous leaf texture, longer and slenderer petioles, orbicular immature leaves and fewer (maximal 4) leaves clustering in a fascicle. However, they exhibit the dichotomously branched thorn pattern of *R. cubensis*, and overall leaf size is smaller when compared to the more typical *R. bahamensis*. Anyhow, molecular data of Hilger & Urquiola 99/20 (B!) indicate that this specimen is distinct from (morphologically also similar, but geographically distant) *R. acanthophora* and *R. cuneata* (Irimia et al. 2015).

#### Notes

Representative specimens examined. — THE BAHAMAS. **Crooked Islands**: in coppice along stone wall in hills NE of Cabbage Hill, 22°46’N, 74°13’W [retroactively inferred], 22 Feb 1975 (♀ fl), Correll 44486 (US!); **Exuma**: on summit of rocky ridge W of Moss Town, beyond High School, 23°34’N, 75°52’W [retroactively inferred], 3 Oct 1980 (♂ fl), Correll & Popenoe 51418 (A! F! MO! US!), 3 Oct 1980 (fr), Correll 51419 (A! US! K! F! F! MO!); **Inagua**: coppice adjacent to air terminal, 20°59’N, 73°40’W [retroactively inferred], 20 Feb 1973 (sterile), Gillis & Proctor 11767 (A!); **Mayaguana**: by the path to Horse Pond, 22°20’N, 72°50’W [retroactively inferred], 28 Nov 1967 (fl), Byrne 429 (A!); **San Salvador**: edge of coppice along Queen's Highway, 24°62’N, 74°27’W [retroactively inferred], 21 Nov 1974 (♂ fl), Correll 43864 (F! F! GH! GH! MO!). — CUBA. **Pinar del Río**: Viñales, Mogote, 22°37’N, 83°43’W [retroactively inferred], 9 Mar 1999 (sterile), Hilger & Urquiola s.n. (B! M!); Havana: Marianao, 23°51’N, 82°30’W [retroactively inferred], 23 Oct 1921 (sterile), Ekman 13359 (G! K! US!).

##### Common names

unknown.

### Rochefortia barloventensis

Irimia & Gottschling

Rochefortia barloventensis ***Rochefortia barloventensis*** Irimia & Gottschling, Phytotaxa 236: 63–68, figs 1–2, tab. 1. 2015.—TYPE: Caribbean, Lesser Antilles, Leeward Islands, France. Guadeloupe, Marie Galante: Fréchy district, near Grelin, on wooded hillside, 15º57’N, 61º17’W [retroactively inferred], elev. 65–125 m (May 25, 1960): G.R. Proctor 21020 (♀ fl, fr) (holotype A! isotype: US-1111601!)

#### Description

Shrubs or small trees 1.5–10.0 m tall, branches spreading, galls absent; indument glabrescent, slightly pubescent when immature, trichomes simple; bark greyish white, longitudinally fissured; thorns 0.5–0.7 cm long, slender, acute, simple, scattered, axillary, alternate, glabrescent. Leaves fasciculate; petiole 0.4–1.5 cm long, glabrescent; blade 1.9–3.4(–6.0) cm long, 1.2–3.0 cm wide, obovate, sometimes circular, coriaceous, primary veins prominent, lateral veins in pairs of 4–5, arching, tertiary veins absent; base cuneate; apex rounded or slightly emarginate; adaxial surface with cystolith-like structures in epidermal cells, mostly glabrous, but sometimes also with scattered, simple, bent trichomes, abaxial surface with scattered trichomes on the midrib, immature leaves sometimes barbellate. Inflorescence axillary and terminal, branching sympodial, branches slender, glabrescent, pedicel 0.20–0.40 cm long. Calyx 0.35–0.45 cm long, coriaceous, hirsute outside, glabrous inside, sometimes with a few clustered trichomes on distal part, lobes 0.30–0.35 cm long, 0.32–0.35 cm wide, obovate, apex rounded to slightly acute, margin strigose. Corolla 0.35–0.40 cm long, yellow through light orange, occasionally greenish (Liogier et al. 32280: MO! US!), membranaceous, glabrous on both surfaces, very rarely with scattered trichomes at distal lobes, tube 0.10–0.20 cm long, funnel-shaped, lobes usually 5 (rarely 4 or 6), 0.30–0.40 cm long, obovate, with 5–6 parallel veins and few glandular trichomes distally. Anthers of male flower 0.30–0.40 cm long, oblong, filaments 0.30–0.33 cm long, adnate to the corolla tube for further 0.05–0.08 cm, pollen present, tricolporate; anthers of female flower 0.12–0.14 cm long, filaments 0.05–0.08 cm long, adnate to corolla tube for further 0.10–0.12 cm, pollen absent. Ovary of male flower subglobose, 0.14–0.20 cm long, stylodia 2, 0.10–0.12 cm long, distally setose, ovules absent; ovary of female flower globose, 0.25–0.30 cm long, style bifid, united for 0.03–0.10 cm at the base, branches 0.25–0.30 cm long, sometimes slightly unequal, stigmas 2, extensively capitate. Fruit 0.50–0.60 cm tall, 0.60–0.70 cm wide, globose, deep orange through vermillion, turning blackish brown at maturity; style accrescent, occasionally persistent; pyrene 0.40–0.43 cm tall, 0.30–0.35 cm wide, 0.20–0.25 cm deep, ovoid, abaxial surface reticulate.

#### Distribution

Restricted to islands of the Lesser Antilles (Guadeloupe, Marie Galante, Montserrat, Martinique) and eastern Puerto Rico (symbol "●" in Fig. 3), in arid coastal forests, on wooded hillsides and mesophyll forests at low altitudes (sea level up to 125 m).

#### Ecology

Flowering Apr–May, Jul, Sep–Oct, Dec; fruiting Dec–Jan, May–Jun.

#### Taxon discussion

*Rochefortia barloventensis* was discovered as a species new to science in the course of the present revision of *Rochefortia* (Irimia and Gottschling 2015). With respect to leaf shapes and sizes, it is a morphologically variable species and is intermediate between *R. bahamensis* and *R. cuneata* exhibiting mature leaves size greater than 3.5 cm (and up to 6.0 cm) long in some specimens from Guadeloupe, Martinique and Puerto Rico. Molecular data indicate the distinctiveness of this new species, whose closest relative is *R. acanthophora* from adjacent Puerto Rico and the Dominican Republic. The two species can be reliably distinguished based on inflorescence morphology (many- *versus* few-flowered) and leaf shape (frequently circular *versus* predominantly obovate). Overall similarity, however, is great with *R. bahamensis* from The Bahamas archipelago and also with *R. cuneata* from Jamaica, but molecular data indicate that these species are not closely related (Irimia et al. 2015). The geographical disjunctions provides further evidence for the distinctiveness of all such species. A collection from eastern Puerto Rico has leaves similar in size of *R. cuneata* or even larger (i.e., 3.5–5.0 cm), but molecular data indicate the correct determination as *R. barloventensis* (Irimia et al. 2015), though morphologically unusual.

#### Notes

Representative specimens examined. — PUERTO RICO. **Fajardo**: in forest, El Convento, 18°19'N, 65°37'W [retroactively inferred], 15 Sep 1981 (fl), Liogier et al. 32280 (MO! US!). — FRANCE. **Guadeloupe**: Grande–Terre, Porte D’Enfer, 16°29'N, 61°26'W [retroactively inferred], 19 Jul 1982 (♀ fl), Barrier 3747 (G! P! P! US!); Îles des Saintes, Terre-de-Bas, 15°51'N, 61°38'W [retroactively inferred], 22 Nov 1986 (♀ fl, fr), Sastre 8265 (P! P!); **Martinique**: Caravelle, Château Dubuc, 14°46'N, 60°53'W [retroactively inferred], 9 Apr 1998 (♂ fl), Sastre 9747 (P!).

##### Common names

"bois vert" in Martinique (noted on Jussieu s.n.: P!), "espino" in Puerto Rico (Liogier de Sereys Allut and Martorell 1999).

### Rochefortia cubensis

Britton & P.Wilson

Rochefortia cubensis ***Rochefortia cubensis*** Britton & P.Wilson, Mem. Torrey Bot. Club 16: 96. 1920.—TYPE: Caribbean, Republic of Cuba. Havana: E of Playa de Marianao (May 31, 1917): León 7228 (fr) (holotype: NY-111153!).
Rochefortia cubensis = *Rochefortia oblanceata* G.Klotz, Revista Jard. Bot. Nac. Univ. Habana 3: 105. 1982.—TYPE: Caribbean, Republic of Cuba. Pinar del Río: La Palma, Loma Peluda de Cajalbana, elev. 200–300 m (Sep 15, 1970): J. Bisse & H. Lippold [Flora Cuba] 18273 (sterile) (holotype: HAJB isotype: JE-5984!).

#### Description

Shrubs 1.0–3.0 m tall or small trees up to 6.0 m tall, prickly, branches arching, short shoot galls occasionally present, 0.8–1.0 cm long; indument glabrescent, immature branches finely pubescent, trichomes simple; bark greyish white through grey brown, with longitudinal crevices; thorns 0.3–0.5 cm long, slender, acuminate, dichotomously branched, numerous, axillary, glabrescent. Leaves fasciculate, dark green; petiole nearly sessile or up to 0.2 cm long, glabrescent; blade 0.4–0.9 cm long, 0.3–0.5 cm wide, elliptic, sometimes obovate, coriaceous, primary veins pinnate, secondary veins 5–9, tertiary veins reticulate; base cuneate, rarely round; apex rounded, occasionally emarginated; adaxial surface with distinct cystoliths in epidermal cells, glabrous or sometimes covered with grey, simple, long trichomes (visible by naked eye) emerging from a cystolith-like structure, abaxial surface mostly glabrous, sometimes with scattered trichomes distally and on midrib. Inflorescence axillary or terminal, flowers usually in clusters of 2 or solitary, pedicel 0.02–0.2 cm long. Calyx 0.35–0.40 cm long, 0.30–0.40 cm wide, coriaceous, glabrous or with few scattered trichomes outside, sometimes hirsute, glabrous inside, strigose at tips, lobes 0.30–0.35 cm long, 0.40–0.45 cm wide, divided at the base, ovate to widely ovate, apex slightly acute. Corolla 0.35–0.40 cm long, pale yellow, membranaceous, glabrous on both surfaces, tube 0.20–0.25 cm long, funnel-shaped, lobes 0.30–0.35 cm long, ovate, slightly ciliate at tips. Male flower unknown; anthers of female flower 0.05–0.07 cm long, oblong, filaments 0.07–0.10 cm long, adnate to corolla tube for further 0.05–0.08 cm, pollen absent. Ovary of female flower globose, 0.25–0.32 cm long, stylodia 2, 0.25–0.35 cm long, filiform, glabrous, ovules present, stigmas cotyliform. Fruit 0.20–0.40 cm tall, 0.30–0.50 cm wide, globose; style accrescent, occasionally persistent, pyrene 0.30–0.40 cm tall, 0.23–0.33 cm wide, 0.13–0.20 cm deep, adaxial surface cutate.

#### Distribution

Cuba and Jamaica (symbol "+" in Fig. 3), on limestone soils and serpentine, in coastal tickets and dry forests at relatively low altitudes (0–600 m). Plants with similarly restricted distributions are otherwise rare (Borhidi 1991 lists 10 taxa at the generic level, each with only a few species).

#### Ecology

Flowering Mar, Jun–Jul; fruiting Mar, Jul–Sep, Dec–Jan.

#### Taxon discussion

*Rochefortia cubensis* is a widely distributed and frequently encountered species in Cuba and Jamaica. It is morphologically similar to, but with respect to molecular sequence data distinct from, *R. acanthophora* occurring on eastward Caribbean islands (Irimia et al. 2015). In Cuba, leaves are distinctly smaller in *R. cubensis* than in *R. acanthophora*, but individuals from Jamaica (e.g., Howard & Proctor 15091: A!, March 462: P!) have leaf sizes in the range of *R. acanthophora* and are therefore hard to distinguish based on morphology. In Cuba, *R. cubensis* occurs sympatrically with three other species, namely *R. bahamensis* (Pinar del Río) and *R. oblongata* and *R. stellata* (eastern Cuba). They can be easily distinguished from *R. cubensis* either because of bigger leaf size (*R. bahamensis*, *R. oblongata*) or the presence of multi-branched trichomes (*R. stellata*). In Jamaica, *R. cubensis* is sympatric with *R. cuneata* only, from which it differs in leaf size and texture (coriaceous *versus* membranaceous) and inflorescence (sessile *versus* longly pedicellate).

With respect to leaf size and shape, Klotz (1982) payed too much attention to interspecific variation within a possible complex of species. He expressed his concept by the description of *R. oblanceata* G.Klotz, geographically restricted to Pinar del Río and distinguished from *R. cubensis* by smaller leaves and narrower shapes. Another population with leaves in this range is found in eastern Cuba (Guantánamo) that Klotz considered likewise a distinct species to be described using the epithet <*urbaniana*>. Having investigated the species over its full distribution, we think that such leaf morphologies are in the intraspecific range of *R. cubensis* without clear correlations to biogeography. Future population genetics studies are necessary to evaluate the specific status of multiple populations of *R. cubensis* and possible morphological correlations.

*Rochefortia cubensis* is distinctive in the smallest leaves found among species of *Rochefortia* and has populations on very poor and serpentine substrates, resulting in radically stunted individuals. Such traits resemble *Bourreria microphylla* Griseb. (likewise Ehretiaceae), and such species are elements of microphyllous plant communities of montane xeromorphic woodland exhibiting large portions of endemics (Borhidi 1991). Many individuals of *R. cubensis* have branched thorns that is otherwise shared with *R. oblongata* and *R. stellata* only. Some individuals (e.g., Ekman 17216: K!, León 14843: GH!, Bisse & Köhler [Flora Cuba] 7663: JE!) exhibit short shoots induced by insects (i.e., galls: Fig. 1B). The phenomenon is shared with *R. oblongata* (Fig. 1A) only. Some individuals of *R. cubensis* (e.g., León 11921: GH!, Clemente 3003: GH!) appear as substrate for plants assigned to *Tillandsia* sp. (Bromeliaceae).

#### Notes

Representative specimens examined. — CUBA. **Camagüey**: Santayana, in palms barren on serpentine, 21°23’N, 77°55’W [retroactively inferred], 3 Jun 1924 (♀ fl), Ekman 19029 (G!); **Guantánamo**: Jauco, S Baracoa, coastal tickets, 20°44’N, 74°20’W [retroactively inferred], Jul 1924 (fl), León 11921 (GH!); **Havana**: Mayabeque: Jibacoa, coastal thickets, 23°14’N, 81°85’W [retroactively inferred], 30 Mar 1929 (sterile), León 13858 (GH!); **Holguín**: Santa Ana in Monte Yoro, Farallons, 22°55’N, 81°35’W [retroactively inferred], 1 May 1860 (fr), Wright 3126 (G! GH! GOET, NY! P! YU!); **Matanzas**: second terrace towards mouth of Matanzas Bay, 23°24’N, 81°34’W [retroactively inferred], 18 Mar 1923 (♀ fl, fr), Ekman 17216 (K!); **Santiago de Cuba**: Baracoa: Imías, in valley of Río Tacre, 16 Sep 1975 (fr), Álvarez de Zayas et al [Flora Cuba] 27508 (JE!). — JAMAICA. **St. Catherine**: Healthshire Hills, Near Salt ponds, 17°53’N, 76°56’W [retroactively inferred], 31 Aug 1908 (fl), Harris & Britton 10515 (F! GH! K! US!); Great Goat Island, E side, 17°52’N, 77°25’W [retroactively inferred], 18 Jul 1906 (fr), Harris 9332 (US!); **St**. **Thomas**: Albion Mountain, elev 200 ft, 17°53’N, 76°35’W [retroactively inferred], 10 Nov 1913 (sterile), Harris 11684 (BM!); **St. Ann**: along the Queen's Highway, 2 miles E of Rio Bueno (Kaiser Nature Preserve area), elev 10–50 ft, 18°28’N, 77°26’W [retroactively inferred], 20 Jan 1958 (fr), Howard & Proctor 15091 (A!); **Portland Bight**: Pigeon Island, 10 miles of Old Harbour Bay, 17°47’N, 77°42’W [retroactively inferred], Apr 1920 (sterile), Maxon & Killip 1720 (F! GH! GH! US!).

##### Common names

“bronce”, “carbonero”, “espuela de caballero” (Span. knight’s spur), sargento (Castell Puchades et al. 2013) in Cuba, “green (heart) ebony” in Jamaica.

### Rochefortia cuneata

Sw.

Rochefortia cuneata ***Rochefortia cuneata*** Sw., Prod. 54. 1788, Fl. Ind. occid. 552–553. 1797.—TYPE: Caribbean, Jamaica. Without precise locality (between 1784 and 1786): O.P. Swartz s.n. (♀ fl) (lectotype, designated here: UPS V-6577!).
Rochefortia cuneata = *Rochefortia ovata* Sw., Prod. 54. 1788, Fl. Ind. occid. 554. 1797.—TYPE: Caribbean, Jamaica. Without precise locality (between 1784 and 1786): O.P. Swartz s.n. (fl) (lectotype, designated here: UPS V-6578!).
Rochefortia cuneata = *Rochefortia acrantha* Urb., Symb. antill. 5: 479. 1908.—TYPE: Caribbean, Jamaica. Trelawny: Troy, elev. 500–600 m (Dec 6, 1904): W.H. Harris 8821 (♀ fl) (lectotype, designated here: NY-111151! isolectotypes: F-174394! GH-97337! US-655778!); elev. 300–400 m (Nov 21, 1905): W.H. Harris 9073^a^ (♀ fl) (syntype: BM-953141!).

#### Description

Shrubs up to 2.5 m tall or small trees up to 8.0 m tall, galls absent; indument variously sericeous through glabrescent, trichomes simple; bark grey light through grey dark or brown, superficial grooves present; thorns 0.7–1.0 cm long, slender, acute, simple or rarely branched, scattered, alternate, axillary or rarely terminal, glabrous. Leaves fasciculate, rarely opposite or alternate; petiole 0.3–0.8 cm long, slender, hirsute or sometimes glabrescent; blade 1.5–3.5(–6.6) cm long, 0.5–3.0(–3.5) cm wide, obovate to widely obovate, occasionally orbicular, membranaceous, primary veins prominent, hirsute, secondary veins 4–11, tertiary veins reticulate; base cuneate or rounded; apex rounded, obcordate, sometimes retuse, rarely cleft; adaxial surface brightly glabrescent, with very rare cystoliths-like structures in epidermal cells, ciliate at tips, trichomes emerging from a swelled cystolith cell giving the impression of undulate leaf margin, abaxial surface brightly rugose, with scattered trichomes. Inflorescence axillary or terminal, branches slender, hirsute to glabrescent, pedicel 0.30–0.35 cm long. Calyx 0.35–0.40 cm long, 0.30-0.45 cm wide, coriaceous, hirsute outside, glabrous inside with scattered trichomes at distal blade, lobes 0.30–0.35 cm long, 0.28–0.40 cm wide, divided from the base, obovate, apex rounded. Corolla 0.40–0.55 cm long, membranaceous, glabrous on both surfaces, tube 0.25–0.30 cm long, funnel-shaped, lobes 0.25–0.35 cm long, obovate, slightly cilliate distally. Male flower unknown; anthers of female flower 0.07–0.08 cm long, oblong, filaments 0.03–0.04 cm long, adnate to the corolla tube for further 0.03–0.04 cm, pollen absent. Ovary of female flower globose, 0.25–0.30 cm long, style divided right from the base, branches 0.18–0.25 cm long, stigma cotyliform. Fruit 0.50–0.60 cm tall, 0.50–0.60 cm wide, globose; pyrene 0.45–0.50 cm tall, 0.25–0.30 cm wide, 0.10–0.13 cm deep, abaxial surface cutate.

#### Distribution

Jamaica, and presumably (with very few collections: Ekman 4145: K!, Leonard & Leonard: 11714 US!) also Haiti (Tortue Island) and Dominican Republic (La Vega province with a single collection: Zanoni 15680: MO!), on limestone soils at sea coast, in wooded and rocky hills (symbol "◊" in Fig. 3), at altitudes between 0–600 m.

#### Ecology

Flowering Mar, Jul, Sep, Nov; fruiting Mar, Jul.

#### Taxon discussion

Across Caribbean islands, *R. cuneata* exhibits amongst the largest leaves that have almost comparable size to those of the mainland species *R. lundellii* and *R. spinosa*. The species is most abundant in Jamaica, from where it was also discovered in the late 18^th^ century. Three collections outside Jamaica (one from Dominican Republic and two from Haiti) are morphologically similar to *R. cuneata*, but all attempts failed to verify the determination by DNA sequence data (Irimia et al. 2015).

Olof P. Swartz’ names were not properly typified before the present revision (Johnston 1949: 128 indicated that the type originates from Jamaica). There are multiple of his gatherings present in different herbaria, but only UPS holds specimens assigned to both names *R. cuneata* and *R. ovata* (and O.P. Swartz was active in Uppsala). We designate explicitely such as type material here, because it is most likely that O.P. Swartz saw it and used it for his descriptions. Whether more material of *R. cuneata* (S-R-5496!) and *R. ovata* (B-W5461! C1-8769! LINN-HS471-2! M-196671! SBT12927!) can be considered original material remains conjecture. Lefor (1968) considered the Willdenow specimen as holotype of *R. ovata*, but his rationale is elusive (and has no taxonomic relevance as his thesis is not effectively published: ICN Art. 30.8.). Swartz (1788) and Swartz (1797) distinguished his two Jamaican species based on differing leaf shapes (i.e., cuneate *versus* ovate), but we consider that they are in the morphological range of a single species.

#### Notes

Representative specimens examined. — JAMAICA. **Cockpit**: NE of Dolphin Head, 18°23’N, 78°10’W (retroactively inferred), 20 Mar 1908 (♀ fl, fr), Harris 10302 (F! GH! K! P! US!); **St. Elizabeth**: Kaiser Bauxite area S of Gutters, near pit 101, 18°05’N, 77°36’W [retroactively inferred], 5 Jul 1955 (♂ fl), Howard & Proctor 14443 (A!); **Near Troy**: 18°14’N, 77°37’W [retroactively inferred], Sep 1906 (♀ fl), Britton 593 (F!); **Negril**: near the Lighthouse, sea coast, in rocky woods, 18°16’N, 78°21’W [retroactively inferred], 10 Mar 1908 (♀ fl, fr), Harris 10232 (BM! F! GH! K! P! US!). — DOMINICAN REPUBLIC. **Santo Domingo**: La Vega, SE of Bonao, Flacombridge Domincana mine operation near Loma La Peguera: Arroyo Hato Viejo bw Loma Larga and Loma Fraser, 29 Jul 1981 (fr), Zanoni 15680 (MO!). — HAITI. **Tortue Island**: Pte Questin, Pte Petit Bois, 20°25’N, 72°46’W [retroactively inferred], 28 May 1925 (sterile), Ekman 4145 (K!); N slope, NW of La Vallée, 20°34’N, 72°56’W [retroactively inferred], 6 Jan 1929 (sterile), Leonard & Leonard 11714 (US!).

##### Common names

"green ebony" and "bois vert" in Jamaica.

### Rochefortia lundellii

Camp

Rochefortia lundellii ***Rochefortia lundellii*** Camp in Lundell, Contr. Univ. Michigan Herb. 7: 47–48. 1942.—TYPE: Belize. El Cayo: road between Arenal and Valentin (Jun, 1936): C.L. Lundell 6167 (♀ fl, fr) (holotype: NY-335693! isotypes: GH-97203! GH-97336! LL-372669! LL-372670! MICH-1111531! MO-152639! S-04-2384! US-1688323!).

#### Description

Lianas or shrubs up to 4.5 m tall or trees up to 10.0 m tall, branches arching, galls absent; indument almost glabrescent at maturity, hirsute when young, trichomes simple; bark whitish grey, grey brown or brown dark, with superficial crevices; thorns 0.6–1.5 cm long, robust, acute and slightly curved at tips, simple, scattered (also on older branches), alternate, axillary, hirsute. Leaves fasciculate; petiole 0.3–2.3 cm long, glabrescent; blade (2.0–)5.0–10.5(–13.0) cm long, 1.5–4.5(–6.0) cm wide, obovate, rarely elliptic, coriaceous, primary and secondary veins prominent, secondary veins 6–9, tertiary veins arching; base cuneate; apex acute, retuse, obcordate, sometimes round or cuspidate; adaxial surface bright, with cystolit-like structures in epidermal cells, glabrous, sometimes with scattered, bent trichomes, abaxial surface shiny, papillate, glabrous, sometime barbellate or rarely with trichomes apically. Inflorescence axillary and terminal, branching sympodial, branches slender, hirsute to glabrous, pedicel 0.20–0.50 cm long. Calyx 0.40–0.45 cm long, 0.45–0.49 cm wide, coriaceous, hirsute outside, glabrous inside, lobes 5 or occasionally 4, 0.35–0.40 cm long, 0.35–0.43 cm wide, very widely ovate, apex obtuse, margin strigose. Corolla 0.35–0.60 cm long, yellow, white, dull white or sometimes greenish (Martínez Salas & Álvarez M. 30817: MO!), fragrant or odourless, membranaceous, glabrous on both surfaces, tube 0.10–0.20 cm long, funnel-shaped, lobes 0.30–0.40 cm long, widely ovate, slightly ciliate at tips. Anthers of male flower 0.25–0.38 cm long, filaments 0.10–0.20 cm long, adnate to the corolla tube for further 0.05–0.06 cm, pollen present (SEM); anthers of female flower 0.12–0.19 cm, filaments 0.08–0.10 cm long, adnate to the corolla tube for further 0.04–0.05 cm, pollen absent. Ovary of male flower subglobose, 0.10–0.12 cm long, stylodia 2, 0.05–0.08 cm long, distally strigose, ovules absent; ovary of female flower globose, 0.20–0.25 cm long, style 0.30–0.45 cm long, united in the proximal part for 0.04–0.12 cm, branches 2, 0.20–0.30 cm long, slightly unequal, glabrous, stigmas 2, cotyliform. Fruit 0.50–1.00 cm tall, 0.40–0.90 cm wide, globose, brightly or blackish red at maturity, later becoming orange, widely ovoid or globose; style accrescent; pyrene 0.60–0.80 cm tall, 0.47–0.50 cm wide, 0.30–0.40 cm deep, abaxial surface reticulate, with 2–3 distinct longitudinal ridges.

#### Distribution

Abundant in Mexico, but also present in adjacent and other countries in Central America (Belize, Costa Rica, Guatemala, Nicaragua), as well as western Cuba (symbol "⬡" in Fig. 3), in perennial and deciduous forests on acidic and calcareous soils from low altitudes up to 1400 m. Borhidi (1991) considers the species (as *R. spinosa*) characteristic for the *Bombacopsi cubensi*–*Thrinacetalia morrisii* plant community at the order level occurring in western Cuba.

#### Ecology

Flowering Jan, Apr–Aug; fruiting Feb–Mar, May–Dec.

#### Taxon discussion

The species has a lianescent habit, which is unusual for *Rochefortia* and for Ehretiaceae as well, and larger stems may bear truly thick and extensive thorns. However, herbarium specimens of *R. lundellii* are morphologically difficult to distinguish from South American *R. spinosa* when growth form is not recorded on the label. Consequently, determination has been more or less arbitrary across both species in the past. Molecular data confirm the distinctiveness between both species (Irimia et al. 2015), but acquisition of sequence data for morphological somewhat divergent (e.g., Costa Rican) population remains a future research task.

*Rochefortia lundellii* is a variable species across its different geographical occurrences, particularly regarding size of leaves and thorns, shape of leaf blades and the apex, but also fruit diameter. Specifically, the Mexican population exhibits the largest mature leaves 6.4–11.0(–13.0) cm long, followed shortly by the Costa Rican population 6.2–10.0(–12.5) cm and the Cuban population 4.8–9.0(–11.0) cm. Other slight differences are observed in thorn size, ranging from 0.7–1.5 cm long in Mexican plants over 0.5–1.0 cm long in Costa Rican plants to 0.3–0.7 cm long in Cuban plants. Some Costa Rican specimens consistently have robust thorns that are slightly curved at tips (the trait is shared with some individuals from Mexico, but also from Venezuela assigned to *R. spinosa*). Leaf apex is usually rounded or emarginated in *R. lundellii*, but some specimens from Costa Rica and Mexico display a cuspidate apex. Fruit diameter ranges within the general *Rochefortia* average values except for Costa Rican specimens having the biggest fruits (1.00–1.20 cm diameter) of all species.

Still in the 60ies of the past century, Lefor (1968) considered *R. lundellii* a rarely collected species with a very restricted geographic range. The species name was even ignored by Sauget y Barbis [Léon] and Liogier de Sereys Allut [Alain] (1957) and Klotz (1982), and they filed Cuban specimens under *R. spinosa* with reference to South America. It is impressive to see how collection efforts by herbaria such as MEXU and MO have the potential to completely revise our view on plant species and their distributions. Today, *R. lundellii* is one of the best known species of *Rochefortia* and well documented over a wide geographic range in multiple herbaria.

#### Notes

Representative specimens examined: — CUBA. **Pinar del Río**: Peninsula de Guanahacabibes, bw Piñatas and Yagales, 21°53’N, 84°22’W [retroactively inferred], 18 Mar 1924 (fr), Ekman 18784 (G! K! US!). — COSTA RICA. **Guanacaste**: Parque Nacional Guanacaste Estación Maritza, 10°57’N, 85°29’W [herbarium label], 1 Jul 1989 (fr), INBiol 107 (MO!); Liberia, P.N. Rincón de la Vieja, Cordillera de Guanacaste, Estación Las Pailas, 10°45’N, 85°20’W [herbarium label], 4 Nov 1993 (fr), Espinoza 629 (B! MO!); Tilarán, P.N. Volcán Tenorio, Cuenca del San Carlos, Sector Rancho Capú, elev 700 m, 10°34’N, 85°34’W [retroactively inferred], 11 Apr 2000 (♀ fl), Chaves 404 (G! MO!). — GUATEMALA. **Petén**: Parque Nacional de Tikal, near El Remate, km 58, lado saliente, 17°10'N 89°38'W [herbarium label], 28 Aug 1970 (fr), Ortíz 1251 (F! MO! NY! US!); Flores, Dos Lagunas, Ixcan río, on Aguas Turbios road, 7 km E, 17°44’N, 89°18’W [retroactively inferred], 10 May 1969 (♂ fl), Contreras 8514 (MO! MO!). — MEXICO. **Campeche**: Calakmul, km 1 entrada a la reserva, 24 May 2001 (fr), Pena-Chocarro et al. 1133 (BM!); Calakmul, 8 km N of Calakmul, 18°08’N, 89°47’W [herbarium label], 13 Jul 1997 (fr), Martínez Salas et al. 27703 (MO!); **Chiapas**: Terán, km 6 carretera Tuxtla Gutiérrez-Chicoasén, elev 640 m, 16°43’N, 93°03’W [herbarium label], 13 Aug 1985 (fr), Palacios 2630 (MO!); Berriozábal, 16°48’N, 93°15’W [retroactively inferred], 24 Sep 1950 (fr), Miranda 6669 (US!); **Hidalgo**: Zimapán, La Majada, 20-25 km NE of Zimapan, 20°50’N, 99°29’W [retroactively inferred], Sep 1981 (sterile), Hernández Magaña & Hernández M. 6535 (MO!); **Quintana Roo**: Othón P. Blanco, 8 km NE of Estero Franco, on road bw Chetumal and La Union, 17°58’N, 88°51’W [herbarium label], 4 Jul 1984 (fr), Cabrera Cano & Cabrera 6650 (MO!); Camino ejido caobitas hacia el campamento X-la' Ha', 18°15'N, 88°00'W [herbarium label], 17 May 1984 (♂ fl), Ucán Ek et al. 3384 (F!); **Nayarit**: San Blas, Islas Marias, parte E de la Isla María Magdalena, caminano hacia el S por la costa, 21°26’N, 106°22’W [herbarium label], 25 Nov 1986 (fr), Chiang Cabrera & Flores Franco 1047 (MO!); **Veracruz**: Apazapan, along road from Baños de Carrizal to Emiliano Zapata, 2–6 km SE of Emiliano Zapata, elev 250–400 m, 19°20’N, 96°38’W [herbarium label], 27 Jun 1980 (♀ fl), Hansen & Nee 7487 (F), 27 Jun 1980 (♂ fl), Hansen & Nee 7488 (F! MO!); Catemaco, vicinity of Hotel Playa Escondida, 10 km N of Sontecomapan, elev 50–100 m, 18°35’N, 95°03’W [herbarium label], 30 Jun 1982 (♀ fl, fr), Nee 22548b (F! G! MO! P! US!); San Andrés Tuxtla, Río Máquina, 200 m from Montepio, 18°38’N, 95°61’W [retroactively inferred], 24 Jul 1984 (fr), Cedillo Trigos 2783 (MEXU, MO!), Minatitlán, 2 km N of Uxpanapa (Pob.12) near Pob. 13; 17°14'N, 94°13'W [herbarium label], 18 Oct 1983 (fr), Wendt et al. 4207 (MO!), Hidalgotitlan, Río Solosúchil, bw Cedillo and Escudra, elev 150 m, 17°16’N, 94°37’W [herbarium label], 8 Nov 1974 (fr), Vázquez Torres 1318 (F!). — NICARAGUA. **El Castillo**: Río El Manú, 200 m N, 11°07’N, 84°21'W [herbarium label], 1 Jun 2005 (fl), Guido 4527 (HULE! MO!).

##### Common names

“palo dulce” in Mexico and “carey de costa” in Cuba.

### Rochefortia oblongata

Urb. & Ekman

Rochefortia oblongata ***Rochefortia oblongata*** Urb. & Ekman in Urb., Ark. Bot. 22A.17: 94. 1929.—TYPE: Caribbean, Republic of Cuba. Santiago de Cuba: near Santiago de Cuba, at Río Aguadores (June 14, 1918): E.L. Ekman 9224 (fr) (holotype: S-04-2385! isotypes: G-236086! GH-97339! K-583494!).

#### Description

Shrubs, short shoot galls occasionally present, 0.7–1.0 cm long; indument glabrous, trichomes simple; bark greyish white, slightly fissured; thorns 0.7–1.1 cm long, relatively robust, acute, simple or dichotomously branched, axillary or terminal, glabrous. Leaves fasciculate; petiole 0.4–1.0 cm long, robust, glabrous; blade 1.1–3.9 cm long, 0.7–1.5 cm wide, oblong-ovate, sometimes elliptic, coriaceous, primary veins very prominent, secondary veins 10–16, tertiary veins absent; base cuneate; apex retuse or obcordate; adaxial surface bright, with numerous cystoliths and simple, bent trichomes emerging from an inflated cystolith basal cell, abaxial surface bright, striate, very rarely with scattered trichomes, midrib and tips with a few trichomes. Inflorescence axillary or terminal, secondarily branched, branches slender, with diffusely scattered trichoms, pedicel 0.50–0.60 cm long. Calyx 0.25–0.30 cm long, 0.28–0.30 cm wide, coriaceous, with scattered trichomes outside, glabrous inside, but with a few scattered trichomes at blade tips, lobes 0.20–0.40 cm long, 0.30–0.35 cm wide, broadly obovate to triangulate, apex obtuse. Flowers at anthesis unknown. Mature fruit unknown; pyrene 0.35-0.40 cm tall, 0.28-0.30 cm wide, 0.20-0.22 cm deep, abaxially completely smooth.

#### Distribution

Endemic to eastern Cuba (symbol "⌾" in Fig. 3), in tidewater flats and dry bushes at 0–300 m altitude.

#### Ecology

Fruiting Jun.

#### Taxon discussion

The species is very similar to Jamaican *R. cuneata*, but differs in having (at least some) thorns branched and also in leaf traits exhibiting numerous cystoliths and peculiar roughness at touch. *Rochefortia oblongata* is sympatric with *R. cubensis* and *R. stellata*, from which it can reliably be distinguished based on leaves and petioles that are both much longer than in the other species. Moreover, the abaxial surface of pyrenes is entirely smooth (*versus* reticulate in *R. cuneata* and all other species of *Rochefortia*).

The species is endemic to Southern Cuba and is known only from five herbarium collections. According to the Red List of Cuban Plants (Berazaín Iturralde et al. 2005), *R. oblongata* is assigned as endangered under IUCN criteria.

#### Notes

Representative specimens examined. — CUBA. **Guantánamo**: Caimanera, W of bay, 19°59’N, 75°91’W [retroactively inferred], 22 Nov 1922 (sterile), Ekman 15748 (K!); Baracoa, N of Loma del Cueros, elev 300 m, 20°54’N, 74°33’W [retroactively inferred], 5 Feb 1976 (sterile), Areces et al. [Flora Cuba] 29684 (HAJB!); Imías, Río Tacre, 20°35’N, 74°31’W [retroactively inferred], 16 Aug 1975 (sterile), Álvarez de Zayas et al. Flora Cuba] 27508 (HAJB!); **Holguín**: Sierra de Nipe: Mayarí Abajo, Loma del Winche, 20°27’N, 75°48’W [retroactively inferred], Mar 1968 (sterile), Bisse & Köhler [Flora Cuba] 6972 (HAJB!).

##### Common names

none.

### Rochefortia spinosa

(Jacq.) Urb.

Rochefortia spinosa ***Rochefortia spinosa*** (Jacq.) Urb., Feddes Repert. 13: 472. 1915. *Ehretia spinosa* Jacq., Enum. syst. pl.: 14. 1760, Select. stirp. amer. hist.: 46, pl. CLXXX 18. 1763. *Lutrostylis spinosa* (Jacq.) G.Don, Gen. Hist. 4: 391. 1838.—TYPE: [illustration] Republic of Colombia. Bolívar: Cartagena: pl. CLXXX 18 in Jacq., Select. stirp. amer. hist. 1763.
Rochefortia spinosa = *Ehretia fasciculata* Kunth in Humb., Bonpl. & Kunth, Nov. gen. sp. 3: 66. 1818. *Crematomia fasciculata* (Kunth) Miers, Ann. Mag. Nat. Hist., ser. 4 3: 312–313. 1869. *Morelosia fasciculata* (Kunth) Kuntze, Revis. gen. Pl. 2: 439. 1891. *Bourreria fasciculata* (Kunth) Gürke in Engl. & Prantl, Nat. Pflanzenfam. 4 (3a): 87. 1893.—TYPE: Bolivarian Republic of Venezuela. Sucre: Cumaná (Sep, without year), A.J.A. Bonpland & F.W.H.A. von Humboldt 92 (♀ fl, fr) (holotype: P-670679!).
Rochefortia spinosa = *Rochefortia jacquinii* Griseb., nom. corr. (ICN Art. 60.12.), Abh. Königl. Ges. Wiss. Göttingen 7: 253. 1857.—TYPE: Republic of Colombia. Cundinamarca: Tocaima (s.d.): J. Goudot s.n. (♀ fl, fr) (holotype: GOET-395! isotype: K!).
Rochefortia spinosa = *Rochefortia fasciculata* Gürke in Engl. & Prantl, Nat. Pflanzenfam. 4 (3a): 89. 1893.—TYPE: Republic of Colombia. Without precise locality at Valle del río Magdalena, elev. 300–700 m (Jan, 1852): J.J. Triana 3744 (♀ fl, fr) (holotype: COL-112389! photo of holotype: US-110770!).

#### Description

Trees up to 10.0 m tall or smaller shrubs 2.0–5.0 m tall, crown arching, galls absent; indument glabrescent, young twigs variously pilose through glabrescent; bark grey light to grey brown, longitudinally fissured, wood light; thorns 0.80–1.0 cm long, robust, somewhat acute, sometimes slightly curved at tips, simple, scattered, alternate, glabrous. Leaves fasciculate, occasionally stipulate (2 deciduous stipules, as stated by Lasser 758: US!), dark green, dark pale, sometimes red when older; petiole 0.2–1.5 cm long, slender, hirsute, rarely glabrous; blade 0.9–10.8 cm long, 0.3–4.5 cm wide, obovate, widely obovate, rarely elliptic, coriaceous, primary veins prominent, secondary veins 5–6, tertiary veins reticulate; base cuneate or sometimes rounded, rarely oblique; apex retuse, rounded, obcordate, emarginate, acute, occasionally mucronulate; adaxial surface glabrous or with scattered trichomes (especially on the midrib and at tips), rugose, with cystotliths, abaxial surface glabrous or with stiff trichomes on the midrib and tips. Inflorescence terminal or axillary, secondarily branched, branches slender, glabrescent, pedicels 0.30–0.40 long. Calyx 0.18–0.25 cm long, coriaceous, hirsute outside, glabrous inside, lobes 0.15–0.18 cm long, 0.10–0.20 cm wide, divided from the base, triangulate, apex slightly acute, margin strigose. Corolla 0.35–0.40 cm long, white, yellowish, orange, rarely green (Ferrari 706: F!), tube funnel-shaped, 0.18–0.20 cm long, lobes 0.20–0.25 cm long, ovate, glabrous on both sides, slightly ciliate at tips. Anthers of male flower 0.15–0.30 cm long, filaments 0.20–0.23 cm long, adnate to the corolla tube for 0.10–0.12 cm, pollen present; anthers of female flower 0.08–0.10 cm long, filaments 0.06–0.10 cm long, adnate to the corolla tube for 0.02–0.03 cm, pollen absent. Ovary of male flower subglobose, stylodia 2, branches 0.07–0.10 cm long, glabrous, with strigose, grey trichomes at tips; ovary of female flower globose, 0.12–0.14 cm long, stylodia 2, 0.18–0.22 cm long, slightly unequal, 2 well developed stigmas, cotyliform. Fruit 0.50–0.80 cm tall, 0.60–0.80 cm wide, globose, green when immature, yellowish reddish through red and dark purple during maturation; style accrescent, not persistent; pyrene 0.40–0.60 cm tall, 0.30–0.40 cm wide, 0.15–0.30 cm deep, with various ridges on the abaxial surface.

#### Distribution

Northern South America (symbol "□" in Fig. 3) in deciduous costal dry forests, in medium and high moist forests, on dry steep cliffs and limestone soils at altitudes between 0–1300 m.

#### Ecology

Flowering Jan, Mar–May, Jul, Sep, Nov; fruiting Feb–Dec.

#### Taxon discussion

*Rochefortia spinosa* is among the species with the largest leaf size after *R. lundellii.* Specimens across different geographical areas are relatively homogenous displaying a low variability of morphological traits (e.g., few collections have leave lengths shorter than 5.5 cm). The species is not sympatric with any other species but morphologically, it resembles *R. lundellii*. It can be distinguished from the latter by the presence of 2 stylodia and generally by the less extensive developed thorns on stems. In the past, *R. spinosa* was one of the most confusing *Rochefortia* species and was believed being distributed across multiple islands of the Caribbean. However, molecular data (Irimia et al. 2015) indicate that the species is restricted to northern South America (primarily Colombia and Venezuela, but also Peru though with few collections available).

#### Notes

Representative specimens examined. — COLOMBIA. **Bolívar**: Cartagena, Tierra Bomba, 10°20’N, 75°32’W, 4 Sep 1989 (fr), Cuadros 4332 (MO!); San Juán de Nepomuceno, Santuario Nacional de los Colorados, 70 km SW of Cartagena, 9°58’N, 75°10’W, 13 Jan 1988 (fl), Gentry et al. 60688 (MO!); **Cundinamarca**: bw Viota and Girardot, elev 320–560 m, 4°26’N, 74°35’W, Aug 1964 (fr), Saravia Toledo 4652 (COL!); **Huila**: La Bodega, Cordillera Oriental, elev 650 m, 3°16'N, 74°54'W, 21 Nov 1944 (fr), Little 8954 (COL! US!); Carretera a San Antonio, El Silencio Quebrada San Roque, elev ± 900 m, 3°21’N, 74°47’W, 28 Sep 1990 (fr), Llanos H & Camacho 1824 (COL!); Villavieja, Desierto de la Tatacoa, 3°13’N, 75°10’W [retroactively inferred], 23 Apr 2003 (♂ fl), Figueroa et al. 246 (COL! COL!); **Magdalena**: Valle del Río Cesare (parte occidental), SW of Los Venados, elev 60 m, 10°N, 73°42’W [retroactively inferred], Feb 1961 (fr), Dugand 5590 (COL! US! US!). — PERU. **San Martin**: Valley of Río Huallaga, 29 km S of Tarapoto, near El Abra, 6°40’S, 76°20’W [herbarium label], 6 Feb 1984 (fr), Gentry & Smith 45027 (MO!); Juan Guerra, elev 720 m, 6°37’S, 76°27’W [retroactively inferred], Dec 1929 (fr), Williams 6874 (F!). — VENEZUELA. **Anzoátegui**: Alrededores de Píritu, 10°13’N, 65°31’W [retroactively inferred], 18 Aug 1965 (fr), Agostini 526 (US!); **Aragua**: Zamora, 4 km N of Villa de Cura, 10°33’N, 67°28’W [retroactively inferred], 23 Mar 1969 (♀ fl, fr), Ferrari & Bunting 713 (F!); **Bolívar**: Kavanayen, elev 1300 m, 5°36’N, 61°30’W [retroactively inferred], 27 May 1946 (♂ fl) Lasser 758 (US!); **Carabobo**: Valencia, elev 400 m, 10°45’N, 68°21’W [retroactively inferred], 14 Sep 1959 (fr), Williams 12198 (F!); **Portuguesa**: Guanare, junction bw rivers Tucupido and Guanare, 9°32’N, 69°49’W [retroactively inferred], 15 Apr 1982 (fr), Utrera & Stergios 152 (MO!), Utrera & Stergios 148 (MO!); **Miranda**, bordeando Laguna Grande, 10°33’N, 66°04’W [herbarium label], 27 May 1981 (fl), Berry 3736 (MO!); **Monagas**: Dos Caminos, via Maturin, 9°34’N, 63°84’W [retroactively inferred], Jul 1969 (♀ fl, fr), Aristeguieta et al. 7162 (F! US!), Aristeguieta et al. 7163 (F! US!); **Sucre**: Guarapoturo, via Chacopata, 10°36’N, 63°39’W [retroactively inferred], 13 Sep 1984 (fl), Cumaná Campos 2458 (MO!); Peninsula de Araya, ca 4 km west of Caimancito, elev 10 m, 10°37’N, 63°57’W, 20 May 1981 (fr), Liesner & González 12160 (MO!); **Trujillo**: Valera, Loma de Moron, 9°19’N, 70°34’W [retroactively inferred], 18 Nov 1922 (♂ fl), Pittier 10718 (US!); **Vargas**, Parroquia Catia la Mar, Escuela Naval, 10°36’N, 67°02’W, 20 Oct 1988 (fr), Ramírez 2576 (MO!); **Yaracuy**: Pena, Cambural, bw Yaritagua and Barquisimeto, elev 350–400 m, 10°07’N, 69°11’W, 8 Nov 2006 (♀ fl), Meier *et al*. 14018 (M!).

##### Common names

“macarao”, “pega paloma” and “tachuelo” in Venezuela and “cruceto macho” and “tatacun o revienta puerco” in Colombia.

##### Economic uses

In Venezuela, the species is occasionally cultivated and forms hedgerows together with guamacho (probably *Pereskia guamacho* F.A.C.Weber: Cactaceae, as noted on Ferrari & Bunting 713: F!).

### Rochefortia stellata

Britton & P.Wilson

Rochefortia stellata ***Rochefortia stellata*** Britton & P.Wilson, Mem. Torrey Bot. Club 16: 96. 1920.—TYPE: Caribbean, Republic of Cuba. Santiago de Cuba: Santiago bay, vicinity of El Morro, coastal thicket, Ensenada Cabanita (Mar, 1912), N.L. Britton, E.G. Britton & J.F. Cowell 12634 (♀ fl, fr) (holotype: NY-111155! isotypes: NY-111156! F-492560! US-1047763!).
Rochefortia stellata = Rochefortia stellata subsp. maisiensis G.Klotz, Wiss. Z. Friedrich-Schiller-Univ. Jena, Math.-Naturwiss. Reihe 29: 471. 1980.—TYPE: Caribbean, Republic of Cuba. Guantánamo: Maisí, Mesa del Chivo (Dec 30, 1959): E.E. Liogier [Alain] & L. Figueiras 7061 (fl) (holotype: HAJB).
Rochefortia stellata = *Rochefortia victoriniana* G.Klotz, Wiss. Z. Friedrich-Schiller-Univ. Jena, Math.-Naturwiss. Reihe 29: 471–472. 1980.—TYPE: Caribbean, Republic of Cuba. Guantánamo: N of Baitiquiri (May, 1968): J. Bisse & E. Köhler [Flora Cuba] 7865 (sterile) (holotype: HAJB isotype: JE-5999!).
Rochefortia stellata = *Rochefortia septentrionalis* G.Klotz, Wiss. Z. Friedrich-Schiller-Univ. Jena, Math.-Naturwiss. Reihe 29: 472, fig. 9. 1980.—TYPE: Caribbean, Republic of Cuba. Holguín: Sierra de Nipe, Mayarí Abajo, Loma de la Bandera (Jun, 1967): J. Bisse & L. Rojas [Flora Cuba] 3983 (fr) (holotype: HAJB isotype: JE-5990!).
Rochefortia stellata = Rochefortia septentrionalis var. cristalensis G.Klotz, Wiss. Z. Friedrich-Schiller-Univ. Jena, Math.-Naturwiss. Reihe 29: 472, fig. 10. 1980.—TYPE: Caribbean, Republic of Cuba. Holguín: Sierra Cristal, loma Saca la Lengua (Apr, 1968): J. Bisse & E. Köhler [Flora Cuba] 6923 (fr) (holotype: HAJB isotype: JE-5998!).
Rochefortia stellata = Rochefortia septentrionalis var. obovata G.Klotz, Wiss. Z. Friedrich-Schiller-Univ. Jena, Math.-Naturwiss. Reihe 29: 472. 1980.—TYPE: Caribbean, Republic of Cuba. Holguín: Sierra de Nipe, Charrasco de la Bandera, elev. 400 m (Apr 19, 1960): E.E. Liogier [Alain] & A.J.B. Acuña Galé 7802 (sterile) (holotype: SV).
Rochefortia stellata = *Rochefortia holguinensis* G.Klotz, Wiss. Z. Friedrich-Schiller-Univ. Jena, Math.-Naturwiss. Reihe 29: 472–473. 1980.—TYPE: Caribbean, Republic of Cuba. Las Tunas: Puerto Padre (May 27, 1931): M. Curbelo s.n. 1931 (♀ fl, fr) (holotype: SV).

#### Description

Shrubs or small trees 3.0–5.0 m tall, galls absent; indument tomentose, especially on young organs, trichomes simple or stellate; bark grey whitish to light brown, longitudinally fissured; 0.7–1.3 cm long, slender, acute, successively branched at 2 or 3 levels, numerous, alternate or terminally of twigs, variously tomentose through glabrescent. Leaves fasciculate; petiole 0.4–0.6 cm long, robust, covered with a dense layer of stellate trichomes; blade 0.6–3.1 cm long, 0.4–2.1 cm wide, elliptic, widely elliptic or sometimes widely ovate, coriaceous, primary veins prominent, secondary veins 5–8, tertiary veins arcuate; base rounded or cuneate; apex rounded, retuse, obcordate, rarely mucronulate; adaxial surface bright, with cystoliths, rugose to almost glabrous or with a few scattered trichomes on the midrib, cilliate at tips, or with long (visible by the naked eye), simple, curved trichomes emerging from an inflated cystolith cell, abaxial surface densely tomentose, occasionally almost glabrous. Inflorescence axillary, flowers in clusters of 2 to 8, pedicels 0.10-0.15 cm long. Calyx 0.20–0.25 cm long, coriaceous, densely stellate outside, ciliate at tips, sometimes with simple trichomes scattered inside towards the distal part, lobes 0.18–0.22 cm long, 0.13–0.15 cm wide, shape triangular, apex acute, occasionally tridentate. Flowers at anthesis unknown. Fruit 0.40–0.50 cm tall, 0.40–0.50 cm wide; style 0.18–0.20 cm long, globose, divided in the proximal part, branches 2, 0.08–0.10 cm long, smaller than fruit, persistent (also the stigmas); pyrene 0.35–0.40 cm tall, 0.18–0.20 cm wide, 0.12–0.13 cm deep, abaxial surface with 5-6 longitudianal ridges.

#### Distribution

*Rochefortia stellata* (symbol "•" in Fig. 3) is endemic to the southern and eastern provinces of Cuba (i.e., Granma, Guantánamo, Holguín, Santiago de Cuba; Borhidi 1991) and occurs in coastal forests and tickets on limestone and serpentine soils as well, between 10–600 m altitude, sympatrically with *R. cubensis* and *R. oblongata*.

#### Ecology

Flowering Mar–Aug, Nov–Dec; fruiting: Apr–Aug.

#### Taxon discussion

*Rochefortia stellata* is the most distinctive species and can be easily distinguished from all other *Rochefortia* species, because of the consistent presence of multi-branched (i.e., stellate) trichomes (Fig. 2) on young twigs, petioles and leaves (on abaxial surface, stellate trichomes are much more abundant and persistent over time, whereas those on the adaxial surface can be lost at maturity). Together with the restricted distribution in eastern Cuba, the species can therefore be reliably identified, even in vegetative stage. Many individuals exhibit branched thorns, which is shared with *R. cubensis* and *R. oblongata* only.

Within his Rochefortia sect. Stellatae, Klotz (1980) distinguished a number of taxa at the species level and below, because he observed morphological variation in correlation with geographic occurrence. Specimens with the smallest leave size and narrowest blade (0.8–1.2 cm long, 0.2–0.3 cm wide) occur mainly in Holguín: Sierra de Nipe (being type localities of *R. septentrionalis* and R. septentrionalis var. obovata) and Sierra Cristal (type locality of R. septentrionalis var. cristalensis), but are also found elsewhere. Plants with scattered stellate trichomes on the upper surface are not confined to Baitiquiri (type locality of *R. victoriniana*), but can be also observed in other Cuban provinces. Generally, density and occurrence of stellate trichomes rather conform to ontogeny than to biogeography, as entire plant organs (e.g., young branches, petioles, calyx lobes, both leaf surfaces) are covered with a dense layer of stellate trichomes in early developmental stages, while older organs have lost this indument, except those on the abaxial leaf surface (possibly as an effective protection against evaporation).

Some individuals of *R. stellata* (e.g., Bucher s.n.: F! Bisse & Köhler 8300: JE!) appear as substrate for plants assigned to *Tillandsia* sp. (Bromeliaceae).

#### Notes

Representative specimens examined. — CUBA. **Granma**: Cabo Cruz, 19°50’N, 77°50’W [retroactively inferred], 26 Jun 1924 (sterile), Ekman 19075 (G! K!); **Guantánamo**: Mesa del Chivo, Maisí, 20°14’N, 74°91’W [retroactively inferred], 19 Aug 1939 (fr), León & Victorin 17108 (GH! US!); Baracoa: Cajobabo, monte seco de la Loma de la Luna, 20°41’N, 74°29’W [retroactively inferred], Jun 1967 (fr), Bisse & Rojas [Flora Cuba] 3404 (HAJB! JE!); **Holguín**: Sierra de Nipe, in carrascales at Río Piloto, 20°28’N, 75°48’W [retroactively inferred], 3 Jul 1924 (fl), Ekman 19171 (F! US!); **Santiago de Cuba**: S of town, 20°08’N, 75°49’W [retroactively inferred], 16 Apr 1917 (fl), Ekman 8597 (K!); S coast, near Juragua Beach, 19°53’N, 75°33’W [retroactively inferred], Aug 1929 (♀ fl, fr), Bucher s.n. (F!); Guamá, 1.5 km W of El Macho, 19°58’N, 76°31’W [retroactively inferred], 29 May 1988 (fr), Álvarez de Zayas et al. [Flora Cuba] 65564 (JE!).

##### Common names

“espino de costa” in Holguín and “carey de costa” in Santiago de Cuba.

## Identification Keys

### Key to the species of *Rochefortia*

**Table:** 

1	Abaxial leaf surface with stellate (i.e., multi-branched) trichomes.	***R. stellata***
–	Abaxial leaf surface with simple trichomes or glabrous.	2
2	Thorns (at least some) branched; plants from Cuba.	3
–	Thorns simple, plants from elsewhere (if from Cuba, then mature leaf length <3.0 cm except *R. lundellii*).	4
3	Mature leaf length < 3.0 cm; flowers sub-sessile; pyrenes abaxially ornamented.	***R. cubensis***
–	Mature leaf length > 3.0 cm; flowers distinctly pedicellate; pyrenes abaxially smooth.	***R. oblongata***
4	Flowers sessile, 1–2 at an individual node.	***R. acanthophora***
–	Flowers distinctly pedicellate.	5
5	Mature leaf length > 8.0 cm.	6
–	Mature leaf length < 6.0 cm.	7
6	Lianas from Central America and westernmost Cuba; style divided in proximal half.	***R. lundellii***
–	Trees and shrubs from elsewhere; 2 stylodia.	***R. spinosa***
7	Leaves (occasionally > 6.0 cm) predominantly membranaceous, mainly obovate.	***R. cuneata***
–	Leaves predominantly coriaceous, very widely obovate to orbicular.	8
8	Plants from The Bahamas and western Cuba, with dense cystolith-like structures adaxially.	***R. bahamensis***
–	Plants from the Leeward Islands and Eastern Puerto Rico, adaxial surface mostly glabrous.	***R. barloventensis***

## Discussion

### Alphabethic list of names today assigned to *Rochefortia* (names to be accepted are in bold face)

*Bourreria fasciculata* (Kunth) Gürke, = ***Rochefortia spinosa*** (Jacq.) Urb.

*Crematomia fasciculata* (Kunth) Miers, = ***Rochefortia spinosa*** (Jacq.) Urb.

*Ehretia acanthophora* DC., ≡ ***Rochefortia acanthophora*** (DC.) Griseb.

*Ehretia fasciculata* Kunth, = ***Rochefortia spinosa*** (Jacq.) Urb. (Lefor 1968)

*Ehretia spinosa* Jacq., ≡ ***Rochefortia spinosa*** (Jacq.) Urb.

*Lutrostylis spinosa* (Jacq.) G.Don, ≡ ***Rochefortia spinosa*** (Jacq.) Urb. (Bentham and Hooker 1876)

*Morelosia fasciculata* (Kunth) Kuntze, = ***Rochefortia spinosa*** (Jacq.) Urb.

***Rochefortia acanthophora*** (DC.) Griseb.

*Rochefortia acrantha* Urb., = ***Rochefortia cuneata*** Sw., *syn. nov*.

***Rochefortia bahamensis*** Britton

***Rochefortia barloventensis*** Irimia & Gottschling

***Rochefortia cubensis*** Britton & P.Wilson

***Rochefortia cuneata*** Sw.

Rochefortia cuneata subsp. bahamensis (Britton) G.Klotz, ≡ ***Rochefortia bahamensis*** Britton

*Rochefortia fasciculata* Gürke, = ***Rochefortia spinosa*** (Jacq.) Urb. (Lefor 1968)

*Rochefortia holguinensis* G.Klotz, = ***Rochefortia stellata*** Britton & P.Wilson, *syn. nov*.

*Rochefortia jacquinii* Griseb., = ***Rochefortia spinosa*** (Jacq.) Urb. (Lefor 1968)

***Rochefortia lundellii*** Camp

*Rochefortia oblanceata* G.Klotz, = ***Rochefortia cubensis*** Britton & P.Wilson, *syn. nov*.

***Rochefortia oblongata*** Urb. & Ekman

*Rochefortia ovata* Sw., = ***Rochefortia cuneata*** Sw., *syn. nov*.

*Rochefortia septentrionalis* G.Klotz, = ***Rochefortia stellata*** Britton & P.Wilson, *syn. nov*.

Rochefortia septentrionalis var. cristalensis G.Klotz, = ***Rochefortia stellata*** Britton & P.Wilson, *syn. nov*.

Rochefortia septentrionalis var. obovata G.Klotz, = ***Rochefortia stellata*** Britton & P.Wilson, *syn. nov*.

***Rochefortia spinosa*** (Jacq.) Urb.

***Rochefortia stellata*** Britton & P.Wilson

Rochefortia stellata subsp. maisiensis G.Klotz, = ***Rochefortia stellata*** Britton & P.Wilson, *syn. nov*.

*Rochefortia victoriniana* G.Klotz, = ***Rochefortia stellata*** Britton & P.Wilson, *syn. nov*.

### Excluded names (validly published names are in bold face)

*Desmophyla aliena* Raf., not validly published (ICN Art. 52.1.), Sylva tellur.: 43. 1838.—TYPE: Bolivarian Republic of Venezuela. Sucre: Cumaná (Sep, without year), A.J.A. Bonpland & F.W.H.A. von Humboldt 92 (♀ fl, fr) (holotype: P-670679!), ≡ ***Rochefortia spinosa*** (Jacq.) Griseb.

*Diplostylis* H.Karst. & Triana (ICN Art. 60.8.), not validly published (ICN Art. 53.1., non ≡ ***Diplostylis*** Sond. = ***Adenocline*** Turcz., Euphorbiaceae), in Triana, Nuev. jen. esp.: 25–26. 1854.—TYPE: *Diplostylis fasciculata* H.Karst. & Triana.

*Diplostylis fasciculata* H.Karst. & Triana, not validly published (ICN Art. 35.1.), Linnaea 28: 433. 1857.—TYPE: see *Rochefortia fasciculata* Gürke, = *Rochefortia spinosa* (Jacq.) Urb.

*Lutrostylis inermis* G.Don, not validly published (ICN Art. 52.1.), Gen. hist. 4: 391. 1838.—TYPE: Bolivarian Republic of Venezuela. Sucre: Cumaná (Sep, without year), A.J.A. Bonpland & F.W.H.A. von Humboldt 92 (♀ fl, fr) (holotype: P-670679!), ≡ ***Rochefortia spinosa*** (Jacq.) Griseb.

***Lutrostylis montevidensis*** (Spreng.) G.Don, Gen. hist. 4: 391. 1838. *Ehretia montevidensis* Spreng., Syst. veg. 1: 647. 1825.—TYPE: Oriental Republic of Uruguay. Montevideo: Montevideo (without date), F. Sellow s.n. (sterile) (holotype: †B), = ***Citharexylum barbinerve*** (Spreng.) Moldenke (Verbenaceae).

Rochefortia acanthophora forma microphylla Griseb., not validly published (ICN Art. 38.1.a), Cat. pl. Cub. 210. 1866.—TYPE: Caribbean, Republic of Cuba. Guantánamo: Mt Toro (May 1, [1861]): Ch. Wright [725] 3126 (sterile) (holotype: GOET-394! isotype: GH-97338!), = ***Rochefortia cubensis*** Britton & P.Wilson. Further material collected by Ch. Wright (NY-111150! G-442332! GH-97338! P-3876535! YU-65231!) should not be considered isotype material (Howard 1988).

***Rochefortia brasiliensis*** Hoffm. in Willd. ex Schult., Syst. Veg. 6: 210. 1820.—TYPE: Federative Republic of Brazil. Without precise locality (1801): J.C. von Hoffmannsegg s.n. 1801 (fl) (holotype: B-W5463! isotype: HAL-135753), identity unclear, but *Rochefortia* does not occur in Brazil. Moreover, the Berlin specimen exhibits a number of bisexual flowers excluding it from *Rochefortia* (and *Lepidocordia* as well).

*Rochefortia transversalis* G.Klotz, not validly published (ICN Arts 10.1., 36.1., 38.1.a), Wiss. Z. Friedrich-Schiller-Univ. Jena, Math.-Naturwiss. Reihe 28: 644, 646, 647. 1979.

## Supplementary Material

XML Treatment for Rochefortia

XML Treatment for Rochefortia acanthophora

XML Treatment for Rochefortia bahamensis

XML Treatment for Rochefortia barloventensis

XML Treatment for Rochefortia cubensis

XML Treatment for Rochefortia cuneata

XML Treatment for Rochefortia lundellii

XML Treatment for Rochefortia oblongata

XML Treatment for Rochefortia spinosa

XML Treatment for Rochefortia stellata

## Figures and Tables

**Figure 2a.**
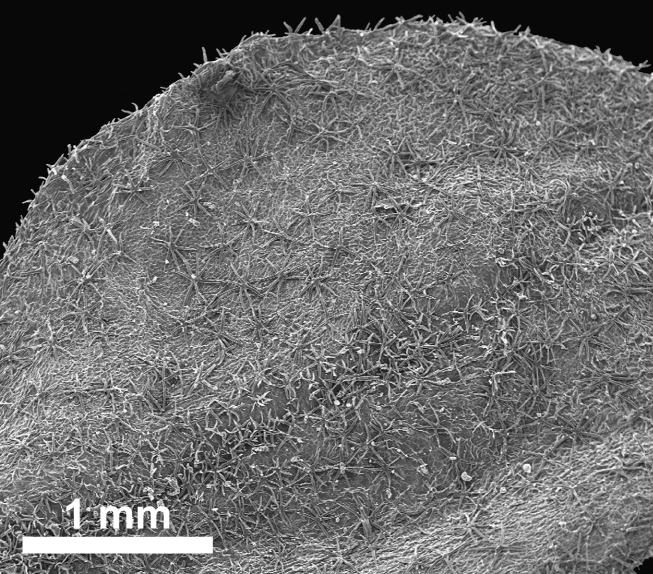


**Figure 2b.**
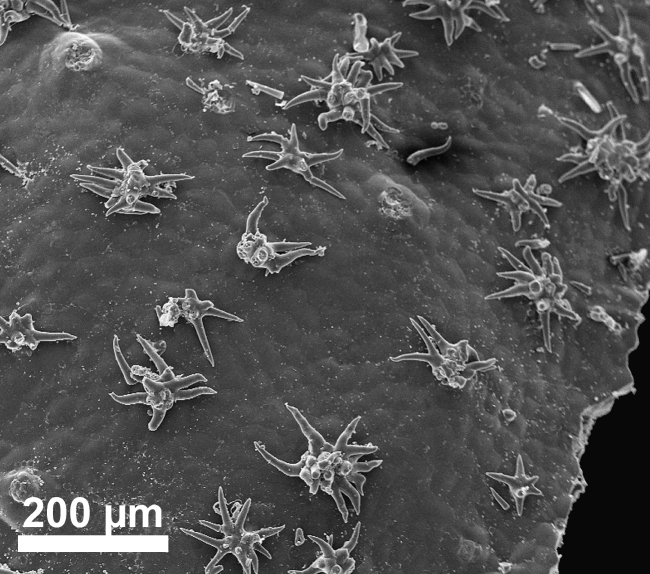


**Figure 2c.**
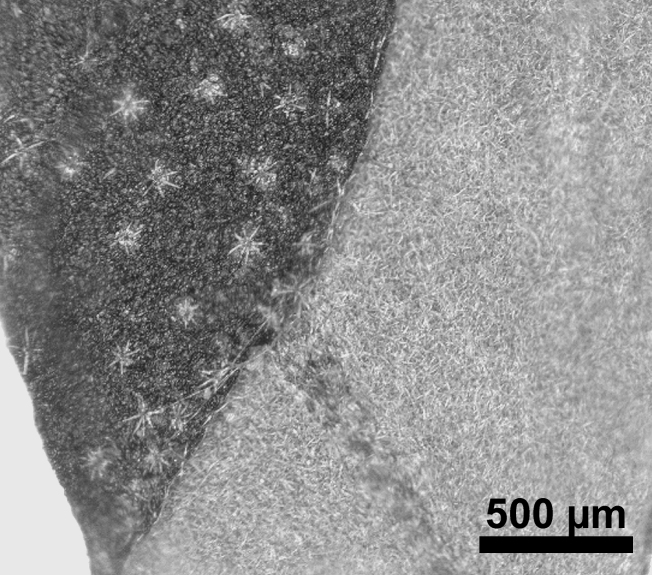


**Figure 2d.**
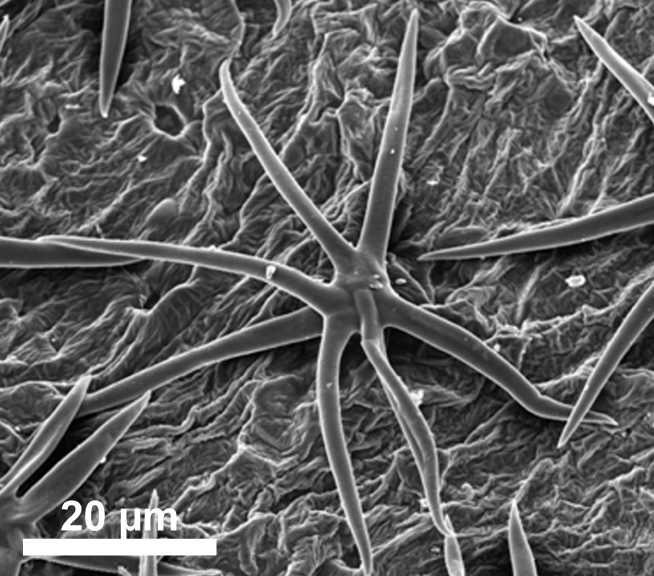


**Figure 2e.**
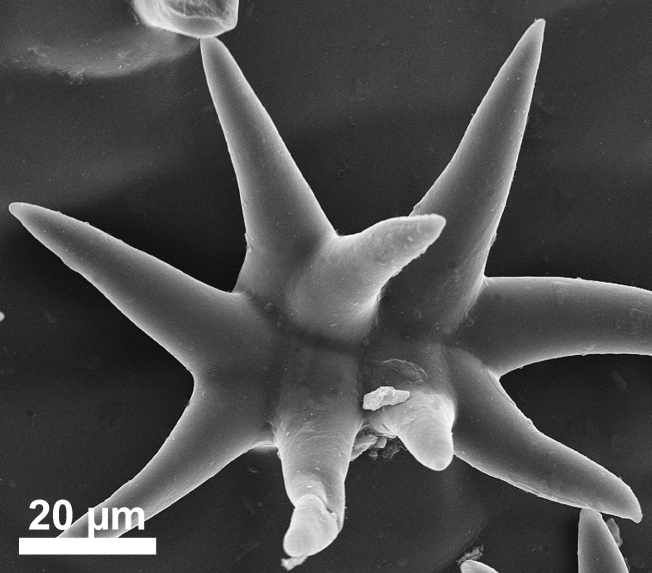


**Figure 2f.**
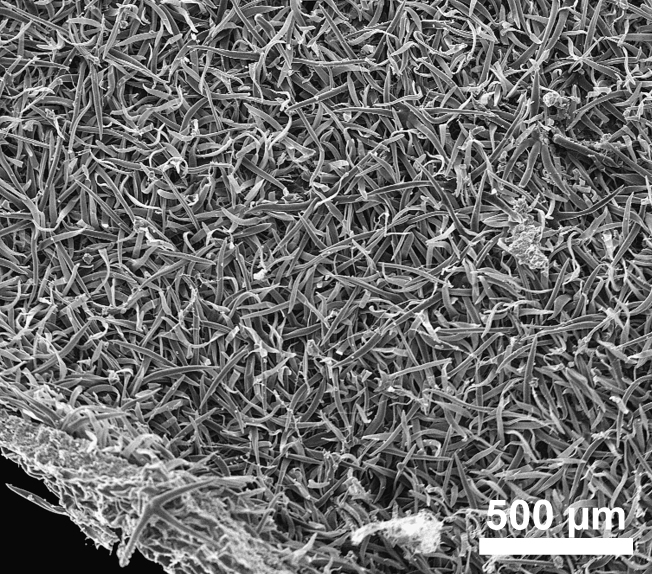


**Table 1.** **Both staminate and pistillate flowers occur contemporary in *Rochefortia*** (pooled specimens of all species inspected in this revision).

	**Jan**	**Feb**	**Mar**	**Apr**	**May**	**Jun**	**Jul**	**Aug**	**Sep**	**Oct**	**Nov**	**Dec**
♂	–	1	3	4	4	3	1	1	–	1	1	–
♀	2	2	6	3	2	7	3	2	–	1	3	3
fruit	5	7	7	4	9	12	12	13	9	7	9	6
